# Multimodal smoking cessation treatment combining repetitive transcranial magnetic stimulation, cognitive behavioral therapy, and nicotine replacement in veterans with posttraumatic stress disorder: A feasibility randomized controlled trial protocol

**DOI:** 10.1371/journal.pone.0291562

**Published:** 2024-09-06

**Authors:** Jonathan R. Young, Carri S. Polick, Andrew M. Michael, Moritz Dannhauer, Jeffrey T. Galla, Mariah K. Evans, Addison Troutman, Angela C. Kirby, Michelle F. Dennis, Claire W. Papanikolas, Zhi-De Deng, Scott D. Moore, Eric A. Dedert, Merideth A. Addicott, Lawrence G. Appelbaum, Jean C. Beckham

**Affiliations:** 1 Department of Psychiatry and Behavioral Sciences, Duke University School of Medicine, Durham, North Carolina, United States of America; 2 VA Mid-Atlantic Mental Illness Research, Education and Clinical Center (MIRECC), Durham, North Carolina, United States of America; 3 Durham VA Health Care System, Durham, North Carolina, United States of America; 4 School of Nursing, Duke University, Durham, North Carolina, United States of America; 5 Duke Institute for Brain Sciences, Duke University, Durham, North Carolina, United States of America; 6 National Institute of Mental Health, National Institutes of Health, Computational Neurostimulation Research Program, Noninvasive Neuromodulation Unit, Experimental Therapeutics & Pathophysiology Branch, Bethesda, Maryland, United States of America; 7 Department of Psychology and Neuroscience, Trinity College of Arts and Sciences, Duke University, Durham, North Carolina, United States of America; 8 Department of Translational Neuroscience, Wake Forest University School of Medicine, Winston-Salem, North Carolina, United States of America; 9 Department of Psychiatry, University of California San Diego School of Medicine, San Diego, California, United States of America; University of Pennsylvania Perelman School of Medicine, UNITED STATES OF AMERICA

## Abstract

Tobacco-related deaths remain the leading cause of preventable death in the United States. Veterans suffering from posttraumatic stress disorder (PTSD)—about 11% of those receiving care from the Department of Veterans Affairs (VA)—have triple the risk of developing tobacco use disorder (TUD). The most efficacious strategies being used at the VA for smoking cessation only result in a 23% abstinence rate, and veterans with PTSD only achieve a 4.5% abstinence rate. Therefore, there is a critical need to develop more effective treatments for smoking cessation. Recent studies suggest the insula is integrally involved in the neurocircuitry of TUD. Thus, we propose a feasibility phase II randomized controlled trial (RCT) to study a form of repetitive transcranial magnetic stimulation (rTMS) called intermittent theta burst stimulation (iTBS). iTBS has the advantage of allowing for a patterned form of stimulation delivery that we will administer at 90% of the subject’s resting motor threshold (rMT) applied over a region in the right post-central gyrus most functionally connected to the right posterior insula. We hypothesize that by increasing functional connectivity between the right post-central gyrus and the right posterior insula, withdrawal symptoms and short-term smoking cessation outcomes will improve. Fifty eligible veterans with comorbid TUD and PTSD will be randomly assigned to active-iTBS + cognitive behavioral therapy (CBT) + nicotine replacement therapy (NRT) (*n* = 25) or sham-iTBS + CBT + NRT (*n* = 25). The primary outcome, feasibility, will be determined by achieving a recruitment of 50 participants and retention rate of 80%. The success of iTBS will be evaluated through self-reported nicotine use, cravings, withdrawal symptoms, and abstinence following quit date (confirmed by bioverification) along with evaluation for target engagement through neuroimaging changes, specifically connectivity differences between the insula and other regions of interest.

## Introduction

In the United States (US), an estimated 28.3 million adults currently smoke cigarettes [1], which causes death or disability in approximately half of this population [2]. There are more tobacco-related deaths than those related to other causes of mortality including acquired immunodeficiency syndrome (AIDS), illicit substance use, alcohol consumption, motor vehicle accidents, suicides and homicides combined [3]. Additionally, individuals with mental illness are disproportionately affected by the medical burden of tobacco use [4]. Smoking rates are especially high among US veterans, and those with posttraumatic stress disorder (PTSD) are more likely to continue smoking despite treatment, increasing the risk of poor health outcomes [5] and reduced life expectancy [6]. Despite substantial progress in developing smoking cessation interventions for veterans with PTSD [7,8], many veterans continue to smoke and are in critical need of innovative treatment options.

The most efficacious strategies found in Veterans Affairs (VA) smoking cessation clinics combine pharmacotherapy with multiple forms of cognitive-behavioral interventions, including self-help materials, group therapy, telephone counseling, and pharmacotherapy, resulting in an abstinence rate of just 23% [9]. Clinic attendance rates at specialty smoking cessation clinics are approximately 13%–14% for all veterans, and those with PTSD are even less likely to follow through on treatment referral [10]. Further, among veterans with PTSD who *do* present to smoking cessation clinics, the efficacy of interventions is even lower [10]. This was demonstrated in the largest smoking cessation trial in veterans with PTSD, with a biochemically verified [bio-verified; exhaled carbon monoxide (CO) < 8 parts per million (ppm) or urine cotinine < 100 nanograms per milliliter (ng/mL)] cessation rate of only 4.5% at 12 months [10]. These findings underscore the urgent need to innovate and develop more efficacious smoking cessation interventions for this clinical population.

Studies evaluating the neurocircuitry of tobacco use disorder (TUD) have found that the insula to be integrally involved [11]. The insula plays a role in the subjective interoceptive awareness of drug craving, thus contributing to motivation to use tobacco and other substances [12]. A seminal study by Naqvi and colleagues showed that individuals with brain lesions involving the insula had drastically improved quit rates and reduced incidence of relapse [13]. Since then, multiple other groups have found correlations between insular involvement and smoking outcomes. A recent study evaluating people who smoke that experienced brain lesions found an addiction remission network that found that lesions positively connected to the insula and cingulate, or negatively connected to the prefrontal cortex, had an increased chance of remission [14]. Similarly, the insula is connected to many regions of the neurobiological craving signature (NCS), a transdiagnostic neuromarker of cravings, that includes the ventromedial prefrontal and cingulate cortices, ventral striatum, temporal/parietal association areas, mediodorsal thalamus and cerebellum [15]. A transdiagnostic “craving network” was also found in a study evaluating functional connectomes, which found anatomical components to include regions in the salience, subcortical, and default mode networks [16]. Since the insula and salience network have been found to be a common neurobiological substance for psychiatric illness there is strong rationale for targeting this network in a population suffering from both TUD and PTSD [17,18].

Repetitive transcranial magnetic stimulation (rTMS) offers a non-invasive method for modulating the activity of targeted brain networks. An rTMS coil (H4-coil, BrainsWay, Israel) designed to broadly stimulate bilateral prefrontal cortices and insula received US regulatory clearance as a short-term smoking cessation aid following a positive multicenter clinical trial [19–21]. Each rTMS session was preceded by a brief provocation procedure to induce nicotine craving. However, most clinical trials (including this pivotal study) on rTMS for smoking cessation have been conducted in civilian patients and have excluded individuals with psychiatric conditions. To improve smoking cessation treatment options for veterans with PTSD, it is critical to evaluate novel brain stimulation methods such as rTMS in this vulnerable population. Furthermore, the development of neuroscience-informed techniques to enhance rTMS, such as neuronavigation based on resting-state functional magnetic resonance imaging (rs-fMRI), is important, allowing clinicians and researchers to not only provide individualized rTMS for smoking cessation but also better understand its mechanisms of action.

## Methods

### Study design

**Fig 1:**
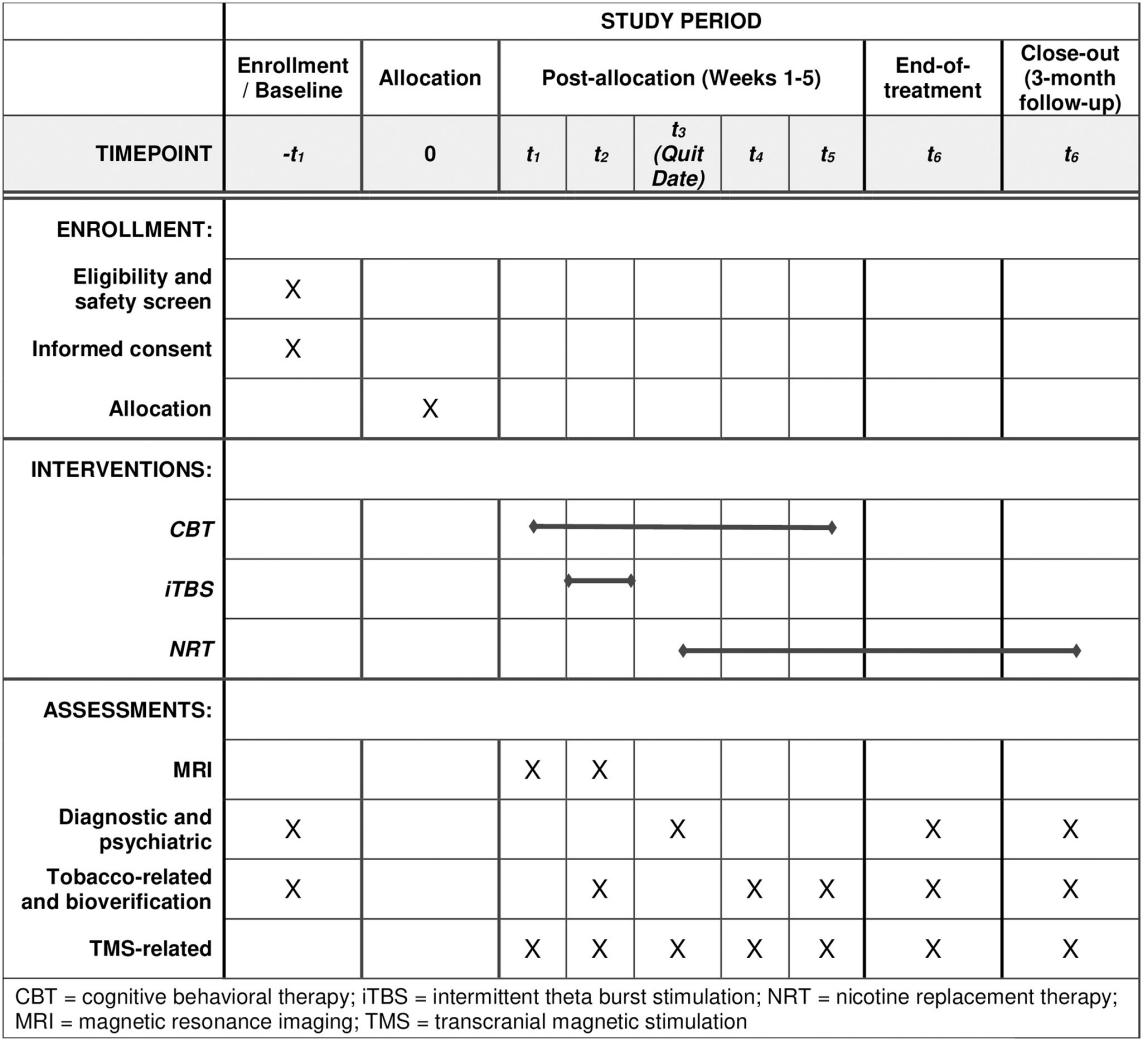
Schedule of study enrollment, interventions, and assessments.

**Fig 2:**
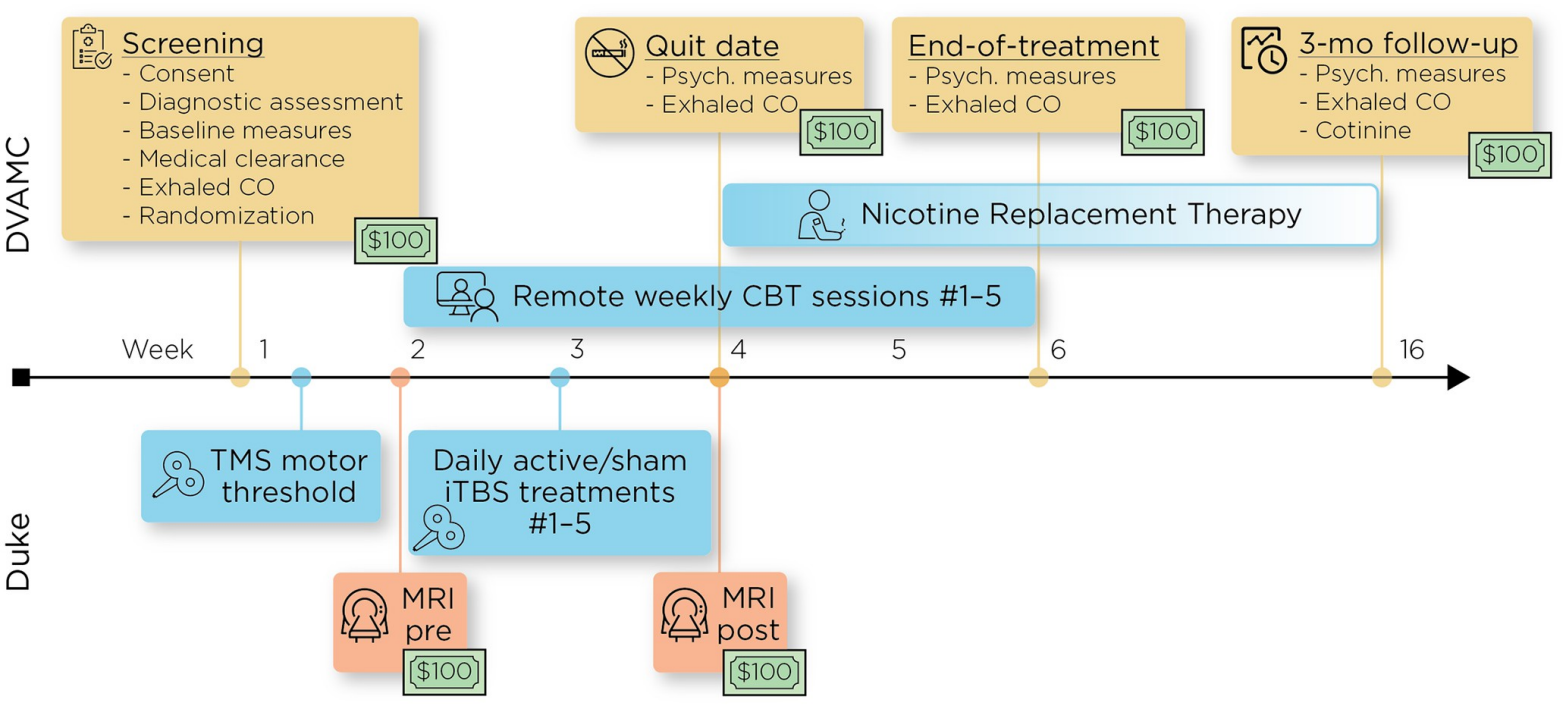
Study process.

The proposed study is a two-arm, parallel design, double-blinded, sham-controlled, randomized trial comparing active- vs sham-iTBS neuronavigated to a region on the post-central gyrus that is functionally connected to the insula (see Figs 1 and 2). Additionally, we will incorporate evidence-based CBT for smoking cessation and NRT. Fifty eligible participants will be randomized to one of two conditions: active-iTBS + CBT + NRT (*n* = 25) or sham-iTBS + CBT + NRT (*n* = 25). Participants will receive baseline structural and functional magnetic resonance imaging (fMRI), twice-daily neuronavigated active or sham (e.g., electrodes placed over right postcentral gyus) iTBS treatments for five consecutive days in the week prior to their intended quit date, followed by a post-treatment MRI. Each iTBS session consists of 1800 pulses delivered at 90% resting motor threshold (rMT) at cortex; the two daily sessions are separated by 50 minutes. All participants will receive 5 weekly sessions of CBT for smoking cessation, starting in the week prior to iTBS. Session 2 occurs during the week of iTBS, and session 3 in the week immediately following iTBS. CBT session 3 is designated as the participant’s intended quit date. All participants will also receive prescriptions of nicotine replacement in the form of 21 mg, 14 mg, and/or 7 mg patches, along with a rescue method of their choice between 2–4 mg nicotine gum or lozenges starting on the quit date and continuing for up to 90 days at individually tapered doses.

The results of this study will address one primary and two secondary aims: (1) to evaluate feasibility and acceptability of the treatment protocol, (2) to estimate effect size of the iTBS intervention, and (3) to demonstrate neural target engagement using fMRI. Feasibility will be determined through recruitment and retention of participants, with a target enrollment of 50 participants and an 80% retention rate (40 completers). Acceptability will be determined by average scores of at least 70% on a 10-point acceptability measure. Efficacy will be evaluated by estimating the effect size via a 7-day point prevalence abstinence at the end-of-treatment and 3-months post-quit date using both self-reported cigarette use and bioverification. Target engagement on fMRI will be determined by evaluating between-group differences on functional connectivity changes between the stimulation target at the sensorimotor cortex and right posterior insula. Additional analyses will evaluate network-level changes on resting-state functional connectivity (rsFC) using the right posterior insula as a region-of-interest; changes in nicotine dependence using the Fagerström Test for Nicotine Dependence (FTND) [22]; changes in nicotine cravings using the Brief Questionnaire of Smoking Urges (QSU-Brief) [23]; changes in nicotine withdrawal on the Minnesota Nicotine Withdrawal Scale-Revised (MNWS-R) [24]; changes in PTSD symptoms as measured by the PTSD Checklist for DSM-5 (PCL-5) [25], changes in depressive symptoms as measured by the Inventory of Depressive Symptoms-Self Report (IDS-SR) [26]; changes in alcohol use as measured by the alcohol use disorders identification test-consumption (Alcohol Use Disorders Identification Test (AUDIT-C) [27]) screening tool; and changes in illicit substance use as measured by the Drug Abuse Screening Test ([28]).

### Participant eligibility criteria and recruitment

Eligible participants will be US veterans, ages 18–75, who meet DSM-5 criteria for tobacco use disorder (TUD) and PTSD diagnoses, have been smoking ≥ 10 cigarettes daily on average over the past 6 months with baseline CO > 6 ppm, are willing to make a smoking cessation attempt, speak and write fluent conversational English, and are appropriate candidates for study procedures (i.e., absence of contraindications for MRI, rTMS, and NRT) (see Table 1 for inclusion and exclusion criteria). Participants may be on stable psychotropic medications, including standard medications for PTSD, but the study team will discourage changes during the trial. To avoid potential confounding effects on smoking cessation as well as potential for modifying seizure risk during rTMS [29,30], veterans who are taking either bupropion or varenicline will be excluded. Participants will be excluded from the study if they have contraindications to rTMS. These include any conditions that increase the risk of seizures, including increased intracranial pressure, neurological conditions (e.g., epilepsy or history of seizures, stroke, space-occupying brain lesions), implanted device or any metal in head or neck except related to dental work; or if they expect to be unstable on their medication regimen during the study. Additionally, we will exclude participants who have a history of myocardial infarction in the past 6 months or other contraindication to NRT; use other forms of nicotine such as cigars, pipes, chewing tobacco or vaping; are pregnant or not willing to use contraception if engaging in heterosexual intercourse (for women of child bearing potential); are currently enrolled in another smoking cessation trial; have major neurocognitive disorder or other reason to not have capacity for informed consent; meet the Structured Clinical Interview for DSM-5 (SCID-5) criteria for a primary psychotic disorder, current mania, or moderate to severe substance use disorder other than nicotine/tobacco in the preceding 3 months; have moderate to severe traumatic brain injury (including history of penetrating head injury, loss of consciousness >20 minutes at time of traumatic injury); require anticonvulsant medications; show encephalomalacia on baseline MRI; or are currently imprisoned or psychiatrically hospitalized. Participants may voluntarily withdraw from participation at any time. The study staff may withdraw a participant one or more of the following reasons: failure to follow the instructions of the study staff, inability to complete the study requirements, or inability to reach participant by telephone after multiple attempts.

**Table 1:** Study eligibility criteria.

Inclusion	Exclusion
US veteran 18–75 years old Meet DSM-5 criteria for current TUD & PTSD Smoking >10 cigarettes/day on average for past 6 months, with carbon monoxide (CO) level > 6 ppm Willing to make cessation attempt Speaks, reads and writes English Willing and able to provide informed consent Has been stable on psychotropic medications for at least three months	Has had a substance use disorder other than tobacco in the preceding 3 months Has a history of myocardial infarction in the past 6 months or has another contraindication to NRT Has a contraindication to TMS or MRI Personal or family history of a seizures or epilepsy History of neurological condition that increases the risk of seizures including stroke or transient ischemic attack, cerebral aneurysm, or severe traumatic brain injury from a penetrating head injury, loss of consciousness > 20 minutes at time of traumatic injury, requiring an anticonvulsant medication for seizures, and/or found to have encephalomalacia on baseline MRI Structural brain lesion, or prior brain surgery Ferromagnetic metal in head (including shrapnel) Implanted devices that may be affected by MRI or TMS (pacemaker, medication pump, cochlear implant, implanted deep brain stimulator) Is pregnant Is unable to complete study procedures Is currently prescribed bupropion and/or varenicline Uses other forms of nicotine such as cigars, pipes, chewing tobacco, or vaping Is unable to provide informed consent due to a major neurocognitive disorder or other reason Meets criteria for a primary psychotic disorder or current manic episode Is currently imprisoned or psychiatrically hospitalized Has previously received rTMS

Participants will be recruited at the Durham VA Health Care System (DVAHCS) through proactive methods (e.g., mailings, flyers) and clinical referrals. Durham, NC, and surrounding areas are heavily populated by veterans. Over 9,000 Afghanistan/Iraq veterans are enrolled at the Durham VA Health Care System, and clinical reminder data indicate that 1 in 3 (32.7%) are currently smoking. Participants will receive reimbursement for their time regardless of study completion, consistent with the extent of participation, up to $600 ($100 per non-treatment session). Study visit payments are to compensate participants for their time and travel expenses.

We will use VA Data Access Request Tracker (DART) requests to identify additional veterans with PTSD and tobacco use disorders. We have used similar procedures to successfully recruit veterans with PTSD in the past. Any potential participant who is identified via a DART request will be sent a recruitment letter by mail or e-mail that provides basic information about the study. The letter will inform veterans that they will be contacted by phone in the coming days regarding their interest in participating in the study. In the letter, potential participants will be given an “opt-out” number to call to decline participation and/or further contact regarding participation. One to two weeks after the mailing, veterans who have not called to decline participation will be called by a study staff member to request their participation in the research study.

If any participant contacts or is contacted by the study coordinator regarding participation, a telephone script will be used to inform them about the study and conduct a preliminary determination of eligibility. If after the telephone screening a participant is considered potentially eligible for participation, he/she will be scheduled for an initial screening session. Once a participant reports to the laboratory to begin the study, the study staff member obtaining consent will explain the study in detail, provide the participant with a DVAHCS Institutional Review Board (IRB)-approved written consent form explaining the procedures and risks, and answer any questions. The initial consent process and documentation takes place either in a quiet, private office at DVAHCS or remotely using DocuSign. Participants will be encouraged to read the consent prior to participation. Participants will also be given a copy of the signed informed consent form and phone numbers to call if they have additional questions about the consent form or the research, if they have any problems during the study, or if they have questions about participating in research studies in general. No study procedures will begin until informed consent has been obtained. Participants deemed to be eligible at DVAHCS will subsequently complete a separate consent form for procedures at Duke University Medical Center (DUMC) (see S1 Appendix for a sample DVAHCS consent form and S2 Appendix for a sample DUMC consent form).

### Sample size and power considerations

Consistent with the aims of feasibility trials, the current study was not designed to have sufficient statistical power to detect statistically significant between-group differences in feasibility variables and smoking cessation outcomes. Instead, sample size was selected to address scientific aims most effectively within the practical considerations including limited budget and cost of the neuroimaging and rTMS procedures. In contrast to the larger sample sizes that are often proposed for feasibility trials using psychotherapy or medication interventions, the cost of procedures and limited budget necessitate smaller sample sizes. Additionally, a review of rTMS studies found that the number of participants tended to be 20 or fewer in most studies [31]. In addition, prior rTMS feasibility trials [32,33], suggested that rTMS procedures could be preliminarily evaluated and developed with a sample size of 25 per group, for a total of 50 participants. We estimate that approximately 120 veterans will be consented and screened, 50 will be enrolled and randomized to a study condition, and a final sample of 40 participants will complete at least 90% of study procedures. The overall goal of the proposed study is to provide broad estimates of feasibility and acceptability. Due to the small sample size, confidence intervals will be constructed around any between-group differences to provide context on the imprecision of any between-group effects and prevent inferences. We targeted a training award for this study that would provide limited funding but would allow for a modest sample size (*n* = 50) that could suggest indicators of threats to feasibility and assist in design of a subsequent robust clinical trial.

### Clinical and laboratory assessments

To improve equity and access, psychiatric assessments will be completed remotely *via* a secure VA virtual platform. Participants will be initially screened for possible eligibility by phone and then receive a psychiatric interview [SCID-5 and Clinician-Administered PTSD Scale for DSM-5 (CAPS-5)]. Safety screening will be completed in person using the TMS Adult Safety Screen (TASS) [34], the Duke MRI Safety Screening form, and the Ohio State University-Traumatic Brain Injury (OSU-TBI) Identification Method-Short Form. Participants will also complete self-report measures to evaluate for nicotine dependence and cravings, depressive symptoms, and PTSD symptoms. Study inclusion and exclusion criteria will be used to determine eligibility. Table 2 outlines study measures at major time points.

**Table 2:** Measures administered at major study time points.

Measure	Time Point			
	Baseline	Quit Date	End-of-treatment	3-Month Follow-up
Sociodemographic and Military Background	X			
MRI Safety Screening	X			
TMS Safety Screening	X			
OSU-TBI	X			
***Diagnostic Assessment and Psychiatric Measures***				
SCID-5	X			
CAPS-5 (past month)	X			
PCL-5 (past month)	X	X	X	X
IDS-SR	X	X	X	X
AUDIT-C	X	X	X	X
DAST (past year)	X			
DAST (past month)	X	X	X	X
***Tobacco-Related Measures and Bioverification***				
FTND	X	X	X	X
MNWS-R	X	X	X	X
Brief Questionnaire on Smoking Urges	X	X	X	X
*Exhaled CO*	X	X	X	X
*Urinary Cotinine*				X
***TMS-Related Measures***				
Credibility/Expectancy Questionnaire	X	X		
TMS Side Effects Scale		X	X	X
Acceptability Questionnaire		X	X	X
Treatment Allocation Guess		X	X	X

MRI = magnetic resonance imaging; TMS = transcranial magnetic stimulation; OSU-TBI = Ohio State University-Traumatic Brain Injury Identification Method-Short Form; Structured Clinical Interview for DSM-5 = SCID-5; Clinician Administered PTSD Scale for DSM-5 = CAPS-5; PTSD Checklist for DSM-5 = PCL-5; Inventory of Depressive Symptoms-Self Report = IDS-SR; Alcohol Use Disorders Identification Test Consumption Screening Tool = AUDIT-C; Fagerström Test of Nicotine Dependence = FTND; Minnesota Nicotine Withdrawal Scale-Revised = MNWS-RS; DAST = Drug Abuse Screening Tool.

Medical clearance for participants to be enrolled will be made by study physicians and staff using the study eligibility criteria. The TASS, Duke MRI Safety and OSU-TBI screening forms will be used to exclude participants in whom it would be unsafe to proceed with MRI or rTMS. Concurrent medications will be documented as per the latest rTMS guidelines [35].

To increase scientific rigor, participants will be asked to provide exhaled breath CO levels at baseline, quit date, end-of-treatment, and at 3-month follow-up visits. Breath CO levels are an effective way of monitoring for cessation, and have an additional positive impact on shaping and inducing smoking abstinence in the first week of a quit attempt [36]. We will use CO monitors for bioverification [including the use of mobile device-based (video) exhaled CO monitoring] to determine smoking abstinence based on a standardized cutoff value of < 5 ppm [37]. This cut-off value has previously been identified as optimal [38], with good sensitivity and specificity among outpatients [39]. In addition, at the 3-month follow-up visits, urinary cotinine will also be collected for more comprehensive bioverification.

### Randomization

There will be two treatment conditions, active-iTBS + CBT + NRT (*n* = 25) and sham-iTBS + CBT + NRT (*n* = 25). Participants will be randomized by a member of the study team who is not directly involved in recruitment or administering study procedures. The sequence will be password-protected so that only the statistician and other study staff who do not have participant contact will have access to the sequence. Randomization will use a simple block design (*n* = 4) to minimize imbalances in group allocation. Participants will be randomized in a 1:1 fashion into one of two groups, which will be allocated to receive either active- or sham-iTBS. All other study staff will remain blinded to treatment conditions throughout study activities. Unblinding will not be permissible unless obligated by the IRB or Data Monitoring Committee (DMC).

## Treatments

### Smoking cessation counseling

Five weekly evidence-based CBT for smoking cessation sessions [10] will be provided by a therapist with at least a Master’s degree in mental health (e.g., psychology, social work), and who is rigorously trained and receiving ongoing supervision. The counseling sessions will be performed remotely via telephone except for the Quit Date session, which will be provided in-person. At each study visit, including counseling sessions, the Timeline Follow-Back (TLFB) method will be used to gather daily reports of smoking [40].

### Smoking cessation pharmacotherapy

All eligible participants will be prescribed NRT [nicotine patch and at least one rescue method (nicotine gum or lozenge)] to begin on the participant’s intended quit date and continue for at least 60 days. NRT will be prescribed by the study physician, who will work with each participant’s primary care physician or psychiatrist to discuss any contraindications. If no contact with the primary care physician can be made, the participant’s health information will be evaluated by the study physician, who will determine medical clearance to participate in the trial. As mentioned above, neither varenicline nor bupropion will be prescribed due to potential for confounding or influencing seizure risk.

### MRI procedures

Participants will undergo an initial MRI scan (pre-iTBS MRI) to obtain structural imaging (T1-weighted scan) and resting state fMRI while eyes are open. Following the last iTBS session, a second structural and functional MRI scan (post-iTBS MRI) will be conducted at least 1 hour and up to 24 hours later. See S3 Appendix for planned MRI sequences and parameters.

The target site for iTBS will be determined for each participant by transforming the location of the right postcentral gyrus cluster that is most positively connected to the posterior insula, using seed-based resting-state functional connectivity (see analysis section for details), into individual anatomical space (using the pre-iTBS anatomical MRI scan), then drawing an 8 × 8 × 8 mm cubed region of interest (ROI) over the cluster location (approximate Montreal Neurological Institute coordinates: 64, -5, 28). The transformed ROI in the individual’s MRI space will be utilized to determine the optimal TMS coil setup [41] that maximizes the induced ROI electric field using the TMS Analysis Pipeline (TAP) software [42]. In more detail, ROI E-field will be maximized perpendicular in direction with respect to the nearest sulci wall. The TAP software also provides the adjusted rTMS intensity (Stokes formula, [43]) to compensate for the difference in anatomical distances between coil being above the motor cortex as well as coil being over the target ROI. For 90% of rMT, TAP determines the adjusted rTMS intensity. The optimal coil placement will then be loaded into Brainsight as the desired coil setup for the TMS coil operator.

### Cue provocation procedure

Immediately prior to the administration of iTBS, a cue provocation procedure will be implemented in which participants watch a 6-minute video of smoking-related images and follow prompts to engage with a cigarette and lighter. Like previous studies using rTMS for the treatment of TUD, activation of neurocircuitry related to addiction by provocation makes it more amenable to modulation [44]. Developed during the pilot project, 60 tobacco-related images including photos of people smoking were extracted from the cross-validated SmoCuDa database of smoking cues [45] based on highest rankings for “urge to smoke” on a 100-point visual analogue scale (overall mean ± SD = 46.75 ± 27.27) as well as images containing persons of color, veterans, and those with common smoking triggers (e.g., alcohol, coffee, multiple people smoking). Instructions to remove a cigarette and lighter out of a plastic bag, place the cigarette on the lips without lighting it, and then put it back into the bag are repeated 4 times. Participants will be asked to complete questionnaires on levels of cravings (100-point visual analogue scale and QSU-Brief) before and after the provocation procedure as well as after iTBS. This procedure is also included at the screening visit for a baseline measure of cue-induced cravings.

### rTMS procedures

Administration of rTMS will be performed using a MagVenture MagPro X100 and a Cool-B65 A/P figure-of-eight coil.

After baseline MRI, individual rMT will be determined and used to calibrate the intensity of iTBS treatments. To determine rMT, electrodes (Neuroline 720, Ambu) will be placed on the first dorsal interosseous (FDI) muscle of the left hand in a belly-tendon montage, and motor evoked potentials (MEPs) will be recorded by an electromyogram (EMG; Power Lab and LabChart) while single-pulse TMS is administered at the motor “hot spot,” defined as the position over the right motor cortex that elicits the greatest MEP in the left first FDI muscle. Resting motor threshold is then determined by the TMS pulse intensity producing on average a MEP of 50 μV peak-to-peak amplitude or a visual MEP in the FDI, using a maximum likelihood estimator (TMS Motor Threshold Assessment Tool, MTAT 2.0) [46].

Using MagVenture’s subject-specific codes assigned at randomization, which the stimulator identifies as active or sham, the operator is instructed whether to flip the rTMS coil. Sham-stimulation electrodes will also be placed on the scalp at the targeted treatment location to provide the sensation of stimulation during sham rTMS. Coil placement will then be guided in real time by a Brainsight neuronavigational system (Rogue Research) which co-registers participants’ pre-determined stimulation target site with the locations of the head and TMS coil.

iTBS will be administered twice daily separated by 50 minutes for 5 consecutive days. iTBS consists of burst of three rTMS pulses at 50 Hz, each pulse with the above-described and distance-adjusted rTMS intensity, bursting at 5 Hz frequency for a 2-second train, with an 8-second inter-train-interval, for 1800 total pulses (approximately 9 minutes duration) per session. A checklist will be used throughout study administration to promote protocol fidelity between study team members.

## Data summary and analyses

Feasibility endpoints have been operationalized to screen for processes that could need improvement in the subsequent trial. Due to the lacking statistical power to detect effects, we do not plan to do significance testing of smoking and feasibility variables. Instead, we will summarize smoking outcomes by treatment group with confidence intervals to underscore the imprecision of any between-groups difference, and we have pre-specified benchmarks for evaluating feasibility outcomes. Feasibility will be measured by evaluating recruitment and retention of participants, with a target enrollment of 50 participant and 80% retention rate (i.e., 40 participants completing at least 90% of study procedures). Acceptability will be measured using a 10-point scale assessing satisfaction of the study procedures (rTMS, CBT, MRI, etc.); the rTMS intervention will be considered acceptable with an average score of ≥ 7/10. Testing of treatment condition masking will take place at the last rTMS, end-of-treatment, and 3-month follow-up visits in which the participant is asked to guess their allocation (active or sham rTMS) and to provide a level of confidence (1-least confident to 10-most confident); the study staff member then also completes this questionnaire.

We will estimate effect size by evaluating the 7-day point prevalence of smoking based on subject self-report and confirmed using exhaled CO level at end-of-treatment and 3-months post-quit date with abstinence rates averaged over all assessments to take advantage of all available data and adjust for within-subjects clustering of data and using previously established methods of self-reported abstinence and bioverification. Non-abstinence will be defined as self-reported smoking (or other tobacco use) for 7 consecutive days or at least once a week for 2 consecutive weeks. Participants will be considered abstinent if they meet certain criteria. For participants taking NRT at the time of follow-up, they will be considered abstinent if they self-report prolonged abstinence and provide a CO reading < 5 ppm. For those not taking NRT, abstinence will be based on 1) self-reported prolonged abstinence, 2) absence of any biochemical samples indicating smoking (CO ≥ 5 ppm or cotinine ≥ 6 ng/mL), and 3) presence of at least one biochemical sample indicating abstinence (CO < 5 ppm and/or cotinine < 6 ng/mL) [47]. Data diagnostics will be performed and addressed as necessary (e.g., non-normal distributions may be transformed). We will calculate mean and standard deviations for continuous variables, and frequency and percentage for categorical/binary variables. We will determine if we meet successful recruitment by enrolling 50 subjects and retention with 40 subjects completing 90% of study procedures (which corresponds to a 20% attrition rate).

Secondary outcomes will include daily number of cigarettes smoked, subjective levels of withdrawal, craving, and urges to smoke, 7-day point prevalence at 3-months post-quit date, as well as smoking relapse, which is defined as smoking 5 or more cigarettes per day for 3 consecutive days. We will evaluate 7-day point prevalence smoking abstinence by calculating a dichotomous outcome variable (abstinent or not abstinent) to determine the proportion of participants with bioverified abstinence at each assessment from quit date through 3 months follow-up. Due to the relatively small sample size and resulting imprecision of any between-groups difference, we will describe the rates of smoking abstinence by calculating the proportion of participants who are confirmed to be abstinent, and we will calculate confidence intervals around the effect size to provide context on the imprecision of the estimate of effect size. Due to the lacking statistical power, we do not plan to do significance testing. A longitudinal analysis using logistic regression models will also be conducted on abstinence across end-of-treatment and 3-month time periods with predictors of treatment arm, time in months (standardized and centered), and the treatment arm-by-time interaction (bioverified smoking abstinence at each time point will be the binary outcome). To be conservative in specifying smoking abstinence, primary summaries of the data will be conducted by coding all missing smoking data as non-abstinent (i.e., missing = smoking), so that participants with missing follow-up data will be assumed to be smoking. Secondary analyses of smoking outcomes will be completed 1) with complete case analysis that censors missing data from the analyses, and 2) using full information maximum likelihood methods to estimate smoking abstinence using SAS proc glimmix to analyze the treatment arm-by-time interaction as a predictor of smoking abstinence at each timepoint [48]. To meet the missing at random assumption, we will evaluate sociodemographic (age, race, biological sex) and baseline clinical variable (nicotine dependence, PTSD symptoms) that are known to predict smoking cessation to determine whether they are also correlated at least 0.5 with missingness at the timepoint being analyzed [49]. If so, we will include the variable as a covariate in the analysis that uses full information maximum likelihood. Analyses will be conducted in R version 4.0.4 and in SAS 9.4. No interim analyses are planned.

Neuroimaging data will be analyzed using FSL [50], CONN [51] and SPM [52] toolboxes. Functional and anatomical data will be preprocessed using a flexible preprocessing pipeline [53] Non brain regions will be removed from the structural images using FSL’s Brain Extraction Tool (BET). The fractional intensity threshold of BET will be adjusted to accurately extract the brain parenchyma. Functional data will be realigned using SPM realign & unwarp procedure [54], where all scans will be coregistered to a reference image (first scan of the first session) using a least squares approach and a six parameter (rigid body) transformation [55] and resampled using b-spline interpolation to correct for motion and magnetic susceptibility interactions. Potential outlier scans will be identified using ART [56] as acquisitions with framewise displacement above 0.9 mm or global BOLD signal changes above 5 standard deviations [57,58], and a reference BOLD image was computed for each subject by averaging all scans excluding outliers. Functional and anatomical data will be normalized into standard MNI space, segmented into grey matter, white matter, and CSF tissue classes, and resampled to 2 mm isotropic voxels following a direct normalization procedure [58,59] using SPM unified segmentation and normalization algorithm [60,61] with SPM’s default tissue probability map. Last, functional data will be smoothed using spatial convolution with a Gaussian kernel of 6 mm full width half maximum (FWHM).

In addition, functional data will be denoised using a standard denoising pipeline [62] including the regression of potential confounding effects characterized by white matter timeseries (5 CompCor noise components), CSF timeseries (5 CompCor noise components), motion parameters and their first order derivatives (12 factors) [63], outlier scans (below 17 factors) [59], session effects and their first order derivatives (2 factors), and linear trends (2 factors) within each functional run, followed by bandpass frequency filtering of the BOLD timeseries [64] between 0.008 Hz and 0.09 Hz. CompCor [65,66] noise components within white matter and CSF will be estimated by computing the average BOLD signal as well as the largest principal components orthogonal to the BOLD average, motion parameters, and outlier scans within each subject’s eroded segmentation masks. From the number of noise terms included in this denoising strategy, the effective degrees of freedom of the BOLD signal after denoising will be estimated [67].

Seed-based connectivity map (SBC) will be estimated characterizing the patterns of functional connectivity with 165 ROIs. Functional connectivity strength was represented by Fisher-transformed bivariate correlation coefficients from a weighted general linear model (weighted-GLM [66]), defined separately for each pair of seed and target areas, modeling the association between their BOLD signal timeseries. To compensate for possible transient magnetization effects at the beginning of each run, individual scans will be weighted by a step function convolved with an SPM canonical hemodynamic response function and rectified.

Regarding safety, we will evaluate for adverse events (AEs) at each study visit. rTMS-specific adverse events will be evaluated at the beginning and end of each rTMS session as well as at follow-up visits using a rTMS Acute Side Effects Questionnaire (Table 2). Side effects to NRT will be assessed at each visit following the initiation of NRT at the Quit Date session. For any specific AE types that occur at least 30% more frequently in the rTMS group, the team of investigators will discuss potential ways to prevent or mitigate those AEs. Suicide risk assessment is conducted at each counseling session. The PI will meet at least weekly with study personnel to discuss enrollment, participation, and any adverse events or unanticipated problems. Regular meetings between investigators and the project manager will allow for ongoing progress reports, including the number of participants currently involved in each study, attrition rates, and scheduled data collection from participants, as well as notification and review of any AEs. Safety monitoring for AEs will be conducted in real time by the PI and/or project manager. The following information about adverse events will be collected: 1) the onset and resolution of the AE, 2) an assessment of the severity or intensity (use existing grading scales whenever possible), 3) an assessment of the relationship of the event to the study (definitely, probably, possibly or not related), and 4) action taken (e.g., none, referral to physician, start or increase concomitant medication). The PI will determine the severity of the event, will assign attribution to the event, and will monitor the event until its resolution. Any adverse events will be reported to the DVAHCS and DUMC IRBs in accordance with their Human Research Protection Program’s Standards of Practice. All research projects conducted at DVAHCS and DUMC are required to have yearly IRB review, including a safety review. Additionally, any changes to the project between review periods must be approved by the appropriate IRB prior to fielding. A VA Clinical Science Research and Development (CSRD) DMC has been assigned to oversee the safety of the study. The DMC is primarily responsible for safeguarding the interests of study participants, assessing the safety and efficacy of trial interventions, and monitoring the progress of the study. The DMC will conduct reviews of the clinical trial and monitor recruitment every 12 months. This DMC serves as an independent advisory group to the CSRD Director and provides recommendations about proceeding with the study initially, continuing the study at subsequent reviews, or stopping the study. The DMC is responsible for identifying mechanisms for the completion of various tasks that will impact the safety and efficacy of study procedures and overall conduct of the study. All Serious Adverse Events (SAEs) and Unanticipated Problem Involving Risks to Subjects or Others (UPIRTSOs) will be submitted to the DMC within 3 business days. AEs will be reported in aggregate within progress reports.

This study, formally titled “Neuroimaging correlates and feasibility of transcranial magnetic stimulation (TMS) to improve smoking cessation outcomes in veterans with comorbid PTSD” (TMS-STOP), has received approvals from the IRBs of DUMC (Pro00107816) and DVAHCS (1625460). The trial began enrollment in January 2024 at a single study site (DVAHCS). Information about enrollment status can be found at ClinicalTrials.gov (NCT05723588). Data will be managed and protected in accordance with the research data and safety standards of practice at DVAHCS and DUMC. To ensure confidentiality, all records will be identified by the participant’s identification number, not by name. All raw hard copy data will be kept in a locked file cabinet in a locked room on DVAHCS property. Data files will be stored in a limited access folder on a secure server to which only study staff members have access. Following the trial, de-identified data will be shared in accordance with data sharing requirements of federally funded research.

## Discussion

This RCT will investigate the feasibility, acceptability, and efficacy of fMRI-guided, accelerated iTBS combined with CBT and NRT protocol among a target subject population of veterans with PTSD. This multimodal intervention is particularly novel as it is the first focal rTMS protocol for smoking cessation targeting the insula through functional connectivity while integrating psychotherapy and NRT. The feasibility of this treatment approach has been investigated in an open-label trial. Preliminary outcomes suggest that this approach is acceptable, feasible, and may suggest efficacy for the veterans with PTSD who have participated thus far [32]. Strengths of this study as compared to our recently completed pilot study are the addition of a sham-control group, modification of rTMS to a more efficient patterned form of stimulation (iTBS), incorporation of an accelerated treatment schedule (twice daily treatments), as well as the inclusion of a 3-month post-quit date follow up visit to evaluate longer-term outcomes.

This protocol offers several innovations to previous studies and could have major implications on the future treatment of veterans with PTSD. The outcomes of this study will provide preliminary evidence that a focal rTMS smoking cessation intervention has evidence for feasibility, acceptability, and efficacy for veterans with PTSD. If successful, further clinical research will be required to replicate the results in an adequately powered phase III RCT. Ultimately, this clinical intervention could be incorporated into smoking cessation and interventional specialty VA clinics as a part of the National VA Clinical rTMS Program. Such a treatment option could enhance available treatments for smoking cessation at VA and elsewhere. Additional studies of this treatment protocol may be adapted to veterans without PTSD, as well as for civilian populations.

This study will use state-of-the-art rTMS techniques: 1) intermittent theta-burst stimulation (iTBS), a patterned form of rTMS that uses triplet bursts at 50 Hz given at 5 Hz, 2) accelerated treatment schedule, 3) individualized fMRI-guided targeting with neuronavigation informed by functional connectivity analysis, and 4) synergism with behavioral and pharmacologic interventions. First, the approved rTMS protocol uses 10 Hz rTMS. Depression studies indicate that iTBS is as efficacious as high frequency rTMS while having the advantage of faster treatment time, offering greater efficiency and potentially greater patient tolerability [68]. Second, the rTMS for smoking cessation protocol approved by the U.S. Food and Drug Administration (FDA) involves once-daily treatment sessions. Accelerated rTMS involves administering rTMS for several sessions per day, which may help produce a more rapid and robust response in a shorter amount of time [69]. Third, the development of neuroscience-informed techniques to enhance rTMS, such as neuronavigation based on rs-fMRI, is critical for individualizing rTMS for smoking cessation and understanding mechanisms of action. Our group has previously demonstrated the ability to non-invasively modulate the activity of deep brain structures, including the insula, by targeting a functionally connected cortical target with rTMS [32]. Finally, combining rTMS with evidence-based CBT and NRT has the potential to further enhance the effectiveness of smoking cessation treatment. Indeed, a recent study found significantly greater abstinence rates when combining H11 coil rTMS and varenicline [70]. To date, collected data suggest that the proposed intervention—neuronavigated rTMS over an individualized target within the right post-central gyrus, informed by rs-fMRI, in combination with CBT and NRT—is both feasible and acceptable for veterans with PTSD seeking smoking cessation [32].

The long-term goal of this study is to optimize smoking cessation for veterans with PTSD by further developing a multimodal smoking cessation intervention that adds innovative rTMS to evidence-based CBT and NRT. To our knowledge, this will be the first study to evaluate the efficacy of neuronavigated rTMS over a region functionally connected to the insula through a multimodal intervention approach combining the evidence-based treatments of cognitive behavioral counseling for smoking cessation and NRT. The timing of this trial is optimal given the national expansion of rTMS clinical services at VA hospitals via the National Clinical rTMS Program. Due to this infrastructure, the proposed treatment approach could ultimately be adopted at VA hospitals throughout the country. Aligning with the shift to improve reproducibility in neuroimaging research, the aim of this protocol paper is to increase transparency regarding study methodology [71].

While this study protocol (S1 File) has important strengths, there are weaknesses. Strict eligibility criteria will allow enrollment of the target patient population of veterans with PTSD. However, as noted in our pilot study, a consequence of this is that only about one-half of the participants we screened were able to fit the stringent eligibility criteria. This study is limited to individuals who smoke cigarettes, and so future studies will need to also evaluate the treatment’s applicability to other forms of nicotine consumption (e.g., vaping). Furthermore, while individualized rTMS targeting using functional connectivity to the insula is novel, the feasibility and cost-effectiveness of these procedures in a clinical setting will need to be further evaluated. It is possible that the technical steps required to utilize this protocol including rsFC determination, optimal coil placement, and navigated rTMS would exceed the capacity of most clinical rTMS programs. It will be important to consider modifications that simplify and automate the technical steps to make it more feasible to translate this intervention into the clinical setting.

Finally, there are many future directions that can be explored following the results of this study. For instance, assuming positive outcomes, we intend to organize a multi-center phase III RCT with sufficient power to conclusively determine efficacy of the intervention. Additionally, further explorations within the parameter space may enhance treatment efficacy. These could include increasing the number of sessions, adjusting rTMS parameters for improved target engagement, or re-evaluating the timing of the various interventions relative to planned quit dates. Furthermore, future studies could investigate other populations in other geographical areas to provide further evidence on the efficacy of our findings, as our current protocol is limited to veterans with PTSD in North Carolina.

## Conclusion

Many veterans struggle with PTSD and TUD. This randomized control trial, guided by a recently completed open-label trial, will help determine feasibility, acceptability, and effectiveness of treating smoking cessation in the context of PTSD with a combination of rTMS, CBT and NRT. Overall, this study (S2 File) fills a critical gap as foundational research to better inform larger studies which may help improve treatment options for individuals with TUD.

## Supporting information

S1 Appendix Sample Durham VA Health Care System Research Consent Form. (DOC)

S2 Appendix Sample Duke University Medical Center Research Consent Form. (PDF)

S3 Appendix Planned MRI sequences and parameters. (PDF)

S1 File IRB-Approved study protocol. (DOC)

S2 File SPIRIT checklist. (DOC)
