# Supplementary material for: Multimodal smoking cessation treatment combining repetitive transcranial magnetic stimulation, cognitive behavioral therapy, and nicotine replacement in veterans with posttraumatic stress disorder: A feasibility randomized controlled trial protocol
Source: PLoS One. 2024 Sep 6;19(9):e0291562. doi: 10.1371/journal.pone.0291562 (PMC11379281; doi:10.1371/journal.pone.0291562)
Supplement: S1 Appendix — (DOC) [file pone.0291562.s001.doc]

| **OVERVIEW AND KEY INFORMATION**  Please read this form carefully. You are being asked to participate in this research study because you smoke and you may have posttraumatic stress disorder (PTSD). This study is voluntary and will include only people who choose to take part. Ask your study doctor or study staff to discuss this consent with you, please ask him/her to explain any words or information that you do not understand. It is important that you understand the information on this form.  The purpose of this study is to test the feasibility of a new treatment for smoking cessation in Veterans who have PTSD and smoke.  Your participation in this study will involve 3 office visits and 6 telehealth visits at the Durham VA, and 7 lab visits at Duke University Medical Center. You will be asked to sign a Duke consent form for that portion of the study. You will receive five sessions of smoking cessation counseling and 2 forms of nicotine replacement therapy (that is, nicotine patch and gum or lozenge). At Duke, you may receive either an investigational treatment called repetitive transcranial magnetic stimulation, or rTMS, or a “sham” treatment that looks and sounds like rTMS but includes no actual treatment. You will also have two functional magnetic resonance imaging (FMRI) scans.  The greatest risks of this study occur with the procedures at Duke. Those risks will be described to you in the Duke consent form. For the VA procedures, the greatest risks are side effects of quitting smoking, such as irritability, craving, and constipation. There are also risks associated with using nicotine replacement therapy, such as dry mouth and skin rashes. |
| --- |

**Why is this study being done?**

The study is being done test the feasibility of an investigational treatment for smoking cessation in Veterans with posttraumatic stress disorder (PTSD). Investigational means that the device we are studying is still being tested in research studies and is not approved by the U.S. Food and Drug Administration (FDA)***.***

Most Veterans with PTSD who are trying to quit smoking use medications and counseling. In this study, we are adding a procedure called repetitive transcranial magnetic stimulation (rTMS) to those treatments.

**How Many People Will Take Part in this Study**

Approximately 120 people will be enrolled in this study at the Durham VA Health Care System.

**How Long will i be in this study?**

Your participation in this study will last about four months.

**What is involved in this study?**

If you agree to participate in this research study you will be asked to sign and date this consent form. You will also be asked to sign a Duke University Medical Center (DUMC) consent form.

You will be asked to attend two study visits to determine if you are eligible to participate. The first study visit will be an in-person or telehealth visit during which you will be interviewed about your traumatic experience(s) and PTSD symptoms. If your interview results indicate that still may be eligible to participate, you will be asked to attend an in-person visit. In that visit, you will be asked to complete some questionnaires about your smoking history, mood, medical history, and alcohol and drug use. These questionnaires will be completed using a survey system called Qualtrics. We will ask you to watch a 6-minute video of smoking-related images while you handle a cigarette and lighter. The goal of this procedure is to produce nicotine cravings so that we can measure if your cravings change over time. You will also be asked to provide a breath sample for CO monitoring, which is a method of measuring cigarette smoking. Female participants of childbearing potential will be asked to conduct a pregnancy test at baseline and prior to Duke procedures. You will be paid $100 for completing this baseline assessment.

If after the first session you are eligible to participate in the study, you will receive five sessions of counseling designed to help you quit smoking. Therapy sessions will occur via a telehealth platform such as VA VideoConnect or WebEx or by phone. You will also be prescribed nicotine replacement therapy, or NRT, which will be started at the quit date. NRT is a type of medication to help you stop smoking. You may be familiar with nicotine patches and nicotine gum – those are forms of NRT. You will be offered two forms of NRT – the patch and what we call a “rescue” method like nicotine gum or lozenges. If there is a medical reason why NRT might not be safe for you, a VA doctor must approve for you to use it. Examples of these types of medical reasons are high blood pressure or pregnancy. If you use NRT, your usual VA prescription co-payments will apply.

After you have started your counseling sessions, you will begin the Duke study procedures. These will include a screening procedure called a functional magnetic resonance imaging, or fMRI. About two weeks after the fMRI, you will come in for the rTMS procedures. After your fMRI procedure, we will use a procedure like flipping a coin to assign you to one of two groups. If you are assigned to the first group, you will receive five sessions of rTMS. If you are assigned to the second group, you will receive “sham” treatment, which looks, sounds, and feels like rTMS, but doesn’t actually provide magnetic stimulation. This is similar to taking a sugar pill, or placebo. The rTMS machine we will be using during this research study is called the MAGPRO X100 by MagVenture. After you have completed rTMS, you will have another fMRI. The Duke consent form will provide more information about these procedures. After you have completed rTMS and the fMRI sessions, your data will be shared from Duke to VA. You will be paid $200 when VA receives your fMRI data from Duke ($100 for each session).

After your final fMRI, you will attend a post-treatment session in-person. We will ask you to provide another breath sample for CO monitoring. You will also be asked to complete some questionnaires. You will be paid $100 for completing this visit.

You will be asked to attend two follow-up visits – one about two weeks after the post-treatment visit, and another about three months after the post-treatment visit. In these visits, will be asked to provide another breath sample and complete some questionnaires. You will be asked to stop taking NRT. At both of these sessions, if you have not been using NRT, we will ask you to provide a urine sample. These samples will be sent to LabCorp for analysis. You will be paid $100 for attending this session.

The study team will ask you if you would like to receive email notifications regarding your study participation. If you would like, we can send you appointment reminders and other study correspondence this way.

While you are in the study, you will be allowed to continue with any treatments you may already be receiving for PTSD. You can also start new treatments for PTSD. Your PTSD treatment will not be impacted by participation in this study.

**What are the risks and discomforts of participating in this research study?**

If you are assigned to group 1, there are risks associated with the FMRI scan and rTMS treatments. MRI scans can cause anxiety in some people. If you have claustrophobia, please let a member of the study team know. The most serious known risk of TMS is convulsions (seizure). TMS procedures are associated with a very low risk of seizures. Out of over 10,000 people given various forms of TMS to date, 16 people (less than 0.2%) have been reported to have had a seizure. TMS can produce a seizure when a series of pulses is given at high power and when repeated series of pulses are given extremely close together. This study will use only levels of TMS that are within safety guidelines. Levels of TMS that fall within the safety guidelines have not been associated with seizure in appropriately screened individuals. No seizures have occurred in normal volunteers with the dosage of TMS used in this study. To minimize this risk, we will medically screen you for any of the known characteristics that could lead to seizure. For example, persons with epilepsy cannot participate in this study. You will be visually monitored during the TMS for any signs of seizure or muscle twitching. In spite of these precautions, there is a chance that you will experience a seizure. Should this occur, emergency facilities are available. If you have a seizure, you may require hospital admission and follow-up neurological evaluation. Having had a convulsion may make it difficult for you to obtain medical insurance, future employment, and to drive. It is not known whether having had one convulsion will make a person more prone to have future seizures. Should you have a seizure caused by TMS in this protocol, we will provide you with a letter documenting that the seizure was experimentally induced.

The most commonly reported side effect of TMS is a "muscle-tension" type headache. We expect that about three out of ten people may experience a headache with the types of TMS

used in this study. We will make every effort to reduce any discomfort. If a headache occurs, it usually starts during or immediately after the TMS and lasts from minutes to hours after TMS. The headache usually goes away with standard over-the-counter pain medications. Muscle twitching, back ache, and neck pain may also occur. You may also experience some discomfort on your head where the coil is held. This is due to contraction of scalp muscles. Numbness of the face lasting for a short time has also been reported in rare instances that may last for several weeks after receiving the procedure. Syncope (fainting) is considered a rare side effect of TMS and has been reported in individuals who faint during blood draws. If you should experience syncope, you will be withdrawn from the study and have your blood pressure monitored until it returns to a healthy level.

If you are assigned to group 1 or group 2, the clicking noises produced by the TMS procedure are loud enough to be damaging to your ears. You will therefore be required to wear earplugs, provided by the study team. Additional side effects considered to be rare in TMS are dizziness, memory impairment, trouble concentrating, and acute mood changes. If these occur, these effects do not last long and will resolve without need for treatment. There may be other risks that are currently unknown. The long-term effects of rTMS are not known.

There are some other risks to participating in the study.

- You may have some discomfort related to quitting smoking. Quitting smoking will cause nicotine withdrawal that may lead to headaches, nausea, irritability, weight gain, difficulty concentrating, poor sleep, increased appetite, anxious or depressed mood, and craving for cigarettes.
- You may have side effects associated with the use of NRT. For instance, you may have some skin irritation with repeated use of the patches. Also, some people report the following symptoms, particularly if they are also smoking while using the patch: dizziness, feeling lightheaded, increased heart rate, increased blood pressure, nausea, nightmares, or vomiting. These side effects vary depending on what type of NRT you use. A study team member will discuss the possible side effects of the NRT that you choose to use.
- There is the potential risk of loss of confidentiality. Every effort will be made to keep your information confidential; however, this cannot be guaranteed.
- Some questions asked as part of this study may make you feel uncomfortable or increase distress. This discomfort or increased distress is usually temporary and well tolerated. You do not have to answer questions and you can take a break at any time. You can call the study team at any time if you experience any discomfort related to the research.
- There is a risk of frustration related to delays that sometimes happen with providing your study payment.

**Will i benefit from taking part in this research study?**

You may benefit from quitting smoking, but this benefit is not guaranteed. You may not personally benefit from taking part in this study, but your participation may lead to knowledge that will help people in the future.

**What other options or Alternatives do i have?**

Taking part in this study is your choice. You may choose to not participate. The VA has smoking cessation clinics that provide counseling and medications to help you quit smoking. You may be eligible to receive smoking cessation treatment in one of those clinics.

**How will my research data be protected and secured?**

Your information used for this study will be kept confidential as required by law. The results of this study may be used for scientific purposes or for publication, but these results will not include any information that would identify you. Your identity will not be disclosed without your consent, or unless required by law. Your research records will be maintained and destroyed according to VHA records retention requirements.

All study data will be kept in a secured file to which only study team members will have access. Hard copy paper records (that is, any forms you sign) will be stored in a locked filing cabinet in the study coordinator’s locked office, within this research lab at the Durham Veterans Affairs Medical Center (DVAMC). Information collected during your telephone sessions will be entered into a computerized database. This database is stored on a VA secured computer server that is password-protected, and only accessible by Dr. Young and his study staff. The key linking code numbers and identifying information will be kept in a locked office in the Durham VA, and will be maintained on password-protected computers behind the VA firewall on the VA secured server.

Your research records may be reviewed by Durham VA staff who are responsible for the safe conduct of this research. We may also provide your research records to federal agencies such as the Office for Human Research Protections (OHRP), the VA Office of the Inspector General (OIG), and the Office of Research Oversight (ORO). We will not share any information with these groups outside the VHA unless they agree to keep the information confidential and use it only for the purposes related to the study. Any information shared with these outside groups may no longer be protected under federal law. These groups may disclose your information to other groups. If the sponsor receives identified information, it is then the sponsor, and not the VA, who is responsible for the security of the information.

As part of this study, de-identified data (that is, data that doesn’t identify) collected from you may be moved to a secured server at Duke University Medical Center. These data will only be available to Dr. Young and/or his study staff. Data will be moved for the purposes of data analysis.

**Certificate of Confidentiality:** To further protect your privacy, this study has been issued a privacy permit to help protect your research records if requested for a court case or for other proceedings.

**Does participation in this research study cost anything?**

Your usual VA prescription co-payments will apply to your NRT prescriptions. There will be no costs to you for any of the other research treatment or research testing done as part of this research study. Some Veterans are required to pay co-payments for medical care and services provided by VA. These co-payment requirements will continue to apply to medical care and services provided by VA that are not part of this study.

**Will i receive any compensation (money or other) for taking part in this research study?**

You will be reimbursed up to $600 for your participation in this study. You will be paid $100 for completing the screening session, $100 for completing the posttreatment visit, and $100 for each of the follow-up visits. You will be paid up to $200 when we receive your FMRI data from Duke. Payment will be made via direct deposit to your bank account, or by check if you do not have a bank account. Money that you receive for participating in research is considered taxable income per Internal Revenue Service (IRS) regulations. The money may be reported to the IRS and you may receive an IRS Form 1099.

**What will happen if i am injured while participating in the research study?**

The VA will provide necessary medical treatment should you be injured by being in this study. You will be treated for the injury at no cost to you. This care may be provided by the Durham VAHCS or arrangements may be made for contracted care at another facility. Every reasonable safety measure will be taken to protect your well-being. You have not released this institution from liability for negligence. In case of research related injury resulting from this study, you should contact your study team. If you have questions about compensation and medical treatment for any study related injuries, you can call the medical administration service at this VA Medical Center at 919-286-6957.

**What Are My Rights to Decline Participation or Withdraw From The Study?**

You can choose to not be in this study, or, if you agree to be in the study, you can withdraw at any time. If you withdraw from the study, no new data about you will be collected for study purposes. We will keep and use the data that we already collected before you withdrew your consent.

If you choose to not be in the study or if you withdraw from the study, there will be no penalty or loss of any benefits to which you are otherwise entitled. This will not affect your relationship with or treatment by the Veterans Health Administration (VHA) or your rights as a VHA patient. You will still receive all the medical care and benefits for which you are otherwise eligible.

**Are there reasons that my research participation may end early?**

Dr. Young may take you out of the study without your consent for one of the following reasons:

1. you have serious side effects or your condition worsens,
2. he decides it is no longer in your best interest to continue in the study,
3. inability to complete study procedures, or
4. failure to follow instructions of investigator and/or study staff.

We will tell you about new information that may affect your health, condition, welfare, or willingness to participate in this study.

**Will the results of this research study be shared with me?**

We do not routinely send out results of the research study. However, if you would like to receive copies of any journal articles that are written using the data we gather during this study, please tell the study coordinator. He/she will make note, and send you a copy of any article about this study.

**Do any of the researchers have a financial interest related to this research study?**

This study is funded by the Department of Veterans Affairs. A part of Dr. Young’s salary is paid for by this research study.

**Where can I find other information about this research study?**

A description of this clinical trial will be available on *http://www.ClinicalTrials.gov*, as required by U.S. Law. This web site will not include information that can identify you. At most, the web site will include a summary of the results. You can search this web site at any time.

**Who do i contact if i have questions or concerns about the research study?**

If you have questions about the research or need to talk to the study team, you can contact Dr. Young at 919-286-0411 Ext. 17-7258 during regular business hours, or contact the Duke University Medical Center operator at 919-684-8111 after hours and ask them to page him. If you have questions about the research or your rights as a research participant, would like to obtain information, offer input, or have other concerns or complaints, you may contact the administrative officer of the research service at (919) 286-0411, extension 177632. If you would like to check that this study is approved by the Durham VAHCS’s Institutional Review Board, please call the research office at (919) 286-6926 or (888) 878-6890, extension 176926.

**Affirmation from participant**

**I have read this form or it has been read to me. My rights as a research participant have been explained to me, and I voluntarily consent to participate in this study. I have received an explanation of what the study is about and how and why it is being done. I authorize the use and disclosure of my identifiable information as described in this form. I will receive a signed copy of this consent form.**

|  |  |
| --- | --- |
| **Signature of Participant** | **Date** |
| **Signature of Person Obtaining Consent** | **Date** |
