# Supplementary material for: Multimodal smoking cessation treatment combining repetitive transcranial magnetic stimulation, cognitive behavioral therapy, and nicotine replacement in veterans with posttraumatic stress disorder: A feasibility randomized controlled trial protocol
Source: PLoS One. 2024 Sep 6;19(9):e0291562. doi: 10.1371/journal.pone.0291562 (PMC11379281; doi:10.1371/journal.pone.0291562)
Supplement: S2 Appendix — (PDF) [file pone.0291562.s002.pdf]

Form
M0345

Duke University Health System

Consent to Participate in a Research Study: Neuroimaging correlates and feasibility of TMS for smoking cessation in veterans with PTSD

CONCISE SUMMARY

The purpose of this study is to determine the feasibility of a non-invasive brain stimulation intervention for helping veterans with posttraumatic stress disorder (PTSD) stop smoking. The intervention is called transcranial magnetic stimulation (TMS).

If you agree to participate in this study, you will be asked to undergo a non-invasive brain scan, magnetic resonance imaging (MRI) before and after a 5-day course of TMS. The MRI scans and TMS protocol will take about two weeks to complete. After that, you will follow up at the VA. The major risks to participating in the study are the remote possibility of TMS causing a seizure, headache, or hearing changes. The MRI may cause you to feel anxious or uncomfortable. We minimize the risks by following procedures that adhere to the latest safety guidelines. You may benefit from stopping smoking. The information that we learn from your participation may help others in the future.

You are being asked to take part in this research study because you because you are participating in a research study at the Durham VA Health Care System (VA). The purpose of the VA study is to test the feasibility of a new intervention for smoking cessation in veterans who have PTSD and smoke. Research studies are voluntary and include only people who choose to take part. Please read this consent form carefully and take your time making your decision. As your study doctor or study staff discusses this consent form with you, please ask him/her to explain any words or information that you do not clearly understand. We encourage you to talk with your family and friends before you decide to take part in this research study. The nature of the study, risks, inconveniences, discomforts, and other important information about the study are listed below.

Please tell the study doctor or study staff if you are taking part in another research study.

Andrew Michael, PhD and Jonathan Young, MD will conduct the study. The Duke portion of the study is funded by Duke University Medical Center, Duke Interdisciplinary Studies Bass Connections Program, and U.S. Department of Veterans Affairs Clinical Science Research & Development Service (1 IK2 CX002610-01).

WHO WILL BE MY DOCTOR ON THIS STUDY?

If you decide to participate, Dr. Jonathan Young will be your doctors for the study and will be in contact with your regular health care provider throughout the time that you are in the study and afterwards, if needed.

Duke University Health System

Consent to Participate in a Research Study: Neuroimaging correlates and feasibility of TMS for smoking cessation in veterans with PTSD

WHY IS THIS STUDY BEING DONE?
The purpose of this study is to test the feasibility of an intervention called repetitive transcranial magnetic stimulation (rTMS) to improve smoking cessation outcomes in veterans with PTSD. Specifically, we are pairing rTMS with a non-invasive brain imaging method, functional magnetic resonance imaging (fMRI). Using fMRI will allow us to accurately target the parts of the brain involved with successful smoking cessation. Only veterans who are participating in the study at the Durham VA will be asked to participate in this Duke study.

HOW MANY PEOPLE WILL TAKE PART IN THIS STUDY?
Approximately 76 people will take part in this study at Duke.

WHAT IS INVOLVED IN THE STUDY?
If you agree to be in this study, you will be asked to sign and date this consent form. You will have the following tests and procedures to make sure that you are eligible:

- Psychiatric screening and medical history (to be completed as part of VA study)
- TMS and MRI safety screening
- Urine screening in women of child-bearing potential to make sure you are not pregnant

If you pass the screening procedures at VA, you will be asked to participate in study procedures there. These study procedures are described to you in the VA consent form. After enrolling in the study, you will be scheduled for an initial brain imaging visit at Duke. In this visit, pictures of your brain will be taken using magnetic resonance imaging (MRI). MRI uses strong magnetic fields and radio waves to make pictures. Special pictures will be taken that provide information about the areas of the brain that become active when you see photos related to smoking. During this part of the study, you will lie on your back on a narrow bed that will be pushed into the MRI machine. The MRI technician will provide padding for your head and knees to make you more comfortable. If you are uncomfortable or feel pain because of lying down, tell the technician immediately. The technician will position your head inside a head tube, and the platform will be pushed into the MRI machine. You will hear a loud noise while the machine is collecting pictures. You will be able to communicate with the technician during the study using a microphone and speaker in the MRI machine. You will hold a squeeze ball in one hand for emergencies, and in the other hand a response pad to answer questions while inside the scanner. The scan time is approximately 60 minutes. You may take a break at any time. You may stop your participation in this study at any time.

Either during this MRI visit or soon thereafter, you will also be introduced to transcranial magnetic stimulation (TMS) to allow you to find out what it is like and for us to individualize the dose of TMS you will be provided during treatment. During this time, the study team will determine the proper strength of TMS to use on you in later visits. This is accomplished by establishing your personal "motor threshold." The motor threshold is a measure of the excitability of the area of the human brain called the motor cortex. To establish this threshold, the study doctor or a member of the study staff will first place TMS coil over the part of your brain that controls the motor activity in your hand. You will hear a

clicking sound and feel a tapping sensation at your scalp. The stimulator will be adjusted to give just enough energy so that the motor region of the brain sends signals to your hand muscles making your hand twitch, which we monitor with electrodes. The smallest amount of energy required to make your hand twitch is called your individual motor threshold. We will also measure hair thickness to further individualize the TMS intensity.

In this study, TMS will be used to temporarily change the way that a part of your brain works. TMS has been approved by the Food and Drug Administration (FDA) as a treatment for depression and obsessive-compulsive disorder. It has also been FDA approved as a short-term smoking cessation aid. In this study, TMS is being used to study if this type of treatment can be used in veterans with PTSD and with the use of investigational tools to help decide where to apply the TMS. The word "investigational" means the study device is still being tested in research studies and is not approved by the U.S. Food and Drug Administration (FDA). To help determine the effect of TMS, you will be randomized, like the flip of a coin, to receive either "active" or "sham" (placebo) TMS, with 50% receiving the active treatment. Sham TMS is provided using an identical coil pointing stimulation away from the brain while two electrodes on the scalp produce the scalp sensation of stimulation without the brain being affected. All participants receive the same set up for the placebo TMS, which consists of small electrodes placed over the treatment location that result in a mild electrical stimulation that does not enter the brain but covers up the sensation of TMS. Both participants and study personnel will be blinded to the randomization.

The TMS equipment consists of an electric stimulator and a wire coil. Turning the stimulator on and off produces brief electrical currents in the coil. These currents create a short-lived magnetic field around that coil (also called a 'magnetic pulse'). The wire coil is coated in plastic in order to insulate the stimulator current. The coil is shaped like an '8', and it is a little larger than a letter-size piece of paper. When the coil is held close to the head, the device generates a magnetic pulse. The pulse can induce very small electric currents in the part of the brain that is closest to the coil. These currents are similar to the currents that the neurons in the brain produce when communicating with each other. By inducing these currents with the TMS coil, we can temporarily change the way that brain region functions, either making the region work more or less.

Prior to each TMS session, you will watch a 6-minute video of smoking-related images and instructions to handle a cigarette and lighter. Dr. Young or a study team member will ask you to provide a cigarette of your favorite brand for this procedure, or one will be provided. The goal of this procedure is to induce cravings for nicotine, which has been found to make the TMS treatment more effective. Questionnaires evaluating levels of cravings and subjective distress will be administered before the video, after the video, and after each TMS session.

The TMS intervention itself consists of two TMS sessions per day taking 9 minutes each separated by 50 minutes. After the first TMS day, you will then take part in four more study visits (two treatments per day) where TMS treatment is provided, as well as a follow-up brain scan. These visits will take approximately 60 minutes each.

Duke University Health System

Consent to Participate in a Research Study: Neuroimaging correlates and feasibility of TMS for smoking cessation in veterans with PTSD

If you agree to participate in this study, a copy of the MRI data will be shared with Dr. Young at the Durham VA. Data will be moved from Duke to VA using an encrypted USB drive.
Participation in this study is voluntary. Your refusal to participate will involve no penalty or loss of benefits to which you are otherwise entitled. Please inform Dr. Michael, Dr. Young or another member of the study team if you decide to terminate your participation in the study. If you do not sign this consent form, you will continue to receive care, but not as a part of this study.

HOW LONG WILL I BE IN THIS STUDY?
Your participation in this Duke study will include up to 8 visits: the first MRI visit (1-2 weeks before TMS), one TMS motor threshold testing session that may occur on the same day as the first MRI, five sessions of TMS (two treatments each) over the course of one week, and a second MRI visit within 24 hours of the last TMS visit. These visits will occur over about a three-week period.

You can choose to stop participating at any time without penalty or loss of any benefits to which you are entitled. However, if you decide to stop participating in the study, we encourage you to talk to your doctor first.

WHAT ARE THE RISKS OF THE STUDY?
MRI Scanning:
There are no known long-term health risks from exposure to the magnetic fields and radio waves used to make MRI brain pictures. However, it is not assured that harmful effects will not be recognized in the future. Strong magnetic fields pose safety risks because they attract metals such as iron, and radio waves can interfere with medical devices such as pacemakers. It can be dangerous for people that have medical devices, metal objects, or metal debris in their bodies to go into a MRI machine. This includes certain dyes found in some tattoos. It is also dangerous to bring loose metal objects into the room containing the MRI machine, because those objects may be pulled towards the magnet and could injure somebody in their way. For these reasons, you will be given an interview and questionnaire prior to this study to make sure that you can be safely scanned. You will be asked to leave all metal objects in lockers provided in the waiting room of the MRI center. You will also be asked to remove any jewelry and articles of clothing with metal inserts or clasps before entering the magnet room. In addition, the MRI scanner makes a loud buzzing noise that could affect your hearing. You will be provided with earplugs and/or headphones in order to protect your hearing. Please ask the study staff or MRI technician if you are unsure about these instructions.

The study involves entering a large room in which a magnet is present. You will be placed on a narrow bed and then slid into a small tunnel approximately 6 feet in length and 25 inches in diameter. You will be asked to lie still for about one hour on this bed. You will hear a loud machine-like noise. You may be asked to have a harmless monitoring device applied during the study. During the study, you can have voice contact and physical contact with someone in attendance if you desire. Some people feel anxious when confined by the small space of the MRI machine. If you feel anxious or uncomfortable inside the

MRI machine, you can tell the study staff over the intercom and you will be removed immediately from the MRI machine.

TMS:
The most serious known risk of TMS is convulsions (seizure). TMS procedures are associated with a very low risk of seizures. Out of over 10,000 people given various forms of TMS to date, 16 people (less than 0.2%) have been reported to have had a seizure. TMS can produce a seizure when a series of pulses is given at high power and when repeated series of pulses are given extremely close together. This study will use only levels of TMS that are within safety guidelines. Levels of TMS that fall within the safety guidelines have not been associated with seizure in appropriately screened individuals. No seizures have occurred in normal volunteers with the dosage of TMS used in this study. To minimize this risk, we will medically screen you for any of the known characteristics that could lead to seizure. For example, persons with epilepsy cannot participate in this study. You will be visually monitored during the TMS for any signs of seizure or muscle twitching. In spite of these precautions, there is a chance that you will experience a seizure. Should this occur, emergency facilities are available. If you have a seizure, you may require hospital admission and follow-up neurological evaluation. Having had a convulsion may make it difficult for you to obtain medical insurance, future employment, and to drive. It is not known whether having had one convulsion will make a person more prone to have future seizures. Should you have a seizure caused by TMS in this protocol, we will provide you with a letter documenting that the seizure was experimentally induced.

The most commonly reported side effect of TMS is a "muscle-tension" type headache. We expect that about three out of ten people may experience a headache with the types of TMS used in this study. We will make every effort to reduce any discomfort. If a headache occurs, it usually starts during or immediately after the TMS and lasts from minutes to hours after TMS. The headache usually goes away with standard over-the-counter pain medications. Neck pain may also occur. You may also experience some discomfort on your head where the coil is held. This is due to contraction of scalp muscles. Numbness of the face lasting for a short time has also been reported in rare instances that may last for several weeks after receiving the procedure. Syncope (fainting) is considered a rare side effect of TMS and has been reported in individuals who faint during blood draws. If you should experience syncope, you will be withdrawn from the study and have your blood pressure monitored until it returns to a healthy level.

The clicking noises produced by the TMS procedure are loud enough to be damaging to your ears. You will therefore be required to wear earplugs, provided by the study team. Additional side effects considered to be rare in TMS are dizziness, memory impairment, trouble concentrating, and acute mood changes. If these occur, these effects do not last long and will resolve without need for treatment. There may be other risks that are currently unknown. The long-term effects of rTMS are not known.

There is also a risk of potential loss of confidentiality. Every effort will be made to keep your information confidential; however, this cannot be guaranteed.

Duke University Health System

Consent to Participate in a Research Study: Neuroimaging correlates and feasibility of TMS for smoking cessation in veterans with PTSD

If you experience any adverse events (a side effect) after leaving the TMS laboratory, please contact Dr. Young by calling 919-286-0411, extension 17-7258, or the study coordinator, Ms. Addison Troutman, at 919-286-0411, extension 17-6702. Should your doctor need to contact Dr. Young, they should call 919-286-0411 and ask the VA operator to contact Dr. Young at home.

For women who could possibly become pregnant
Smoking during pregnancy is associated with an increased risk of complications, including miscarriage, poor fetal growth, preterm birth, and stillbirth. Whether TMS increases these risks further is unknown. If you are a woman who could possibly become pregnant (you have not completed menopause, or you have not had a hysterectomy and/or both tubes and/or both ovaries removed) and you have a partner who is able to father children), a urine pregnancy test will be performed prior to each scan and it must be negative in order to continue.

Although there are no risks to a pregnancy other than those associated with smoking in between scans, if you do become pregnant during the study you will not be able to continue. You and your partner should either abstain completely from vaginal intercourse until after your last scan, or use an effective method of birth control for the same length of time. Dr. Young will review birth control methods with you to make sure that it is appropriate for your medical condition and the level of effectiveness needed for this study.

ARE THERE BENEFITS TO TAKING PART IN THE STUDY?
If you agree to take part in this study, there may be direct medical benefit to you. You may benefit from the TMS treatments by improving your ability to quit smoking. We hope that in the future the information learned from this study will benefit other people with PTSD.

The MRI portion of this study is not a diagnostic medical test and will be of no direct benefit to you, except that it may allow for more precise targeting of the TMS treatments.

Incidental MRI Findings: Medical specialists will not examine the research brain pictures. If you believe that you require a diagnostic MRI test, you should discuss your concerns with your doctor. The brain pictures obtained during this study are for research only and are not designed to search for any existing brain abnormalities. The study staff at Duke is not responsible for a failure to find any existing brain abnormalities. However, in the unlikely event that the technician or study staff collecting the scans notices something that appears abnormal, the technician will ask your permission to obtain an additional set of brain pictures that will be shown to a medically trained expert for clinical evaluation. The results of this evaluation will be provided to you at no charge. The decision to proceed with further examination or treatment based upon this evaluation lies solely with you. You will be responsible for any treatment that you undertake based upon this evaluation.

Duke University Health System

Consent to Participate in a Research Study: Neuroimaging correlates and feasibility of TMS for smoking cessation in veterans with PTSD

WHAT ALTERNATIVES ARE THERE TO PARTICIPATION IN THIS STUDY?

Instead of being in this study, you have the following alternatives: receiving standard of care including medications and talk therapy for smoking cessation with your doctor and/or with the Durham VA Health Care System's specialty smoking cessation clinic. Please talk to your doctor about these and perhaps other options.

WILL MY INFORMATION BE KEPT CONFIDENTIAL?

Participation in research involves some loss of privacy. We will do our best to make sure that information about you is kept confidential, but we cannot guarantee total confidentiality. Your personal information may be viewed by individuals involved in this research and may be seen by people including those collaborating, funding, and regulating the study. We will share only the minimum necessary information in order to conduct the research. Your personal information may also be given out if required by law.

As part of the study, your records may be reviewed in order to meet federal or state regulations. Reviewers may include the Duke University Health System Institutional Review Board, the Food and Drug Administration, and others as appropriate. If any of these groups review your research record, they may also need to review your entire medical record.

After you complete the Duke procedures, one of our study staff members will pull the MRI data from Duke University secured server and a copy will be transferred from Duke to the Durham VA Medical Center. Data will be transferred using an encrypted USB. If you choose to be in this study, data collected from you that can be used for future research will be stored long-term in a two repositories following the completion of the study. The first repository is called ENIGMA, https://enigma.ini.usc.edu/. The second repository is with Omniscient Technologies, o8t.com. Any personal information that could identify you will be removed or changed before files are stored in these repositories for use by other researchers or results are made public. The removal of this information allows your data to be used without anyone knowing which person in the study it comes from. As an additional layer of protection, the repository where the data will be stored requires researchers to formally apply to use the data and obtain approval prior to gaining access.

The study results will be retained in your research record for at least six years after the study is completed. At that time either the research information not already in your medical record may be destroyed or information identifying you will be removed from such study results at DUHS. Any research information in your medical record will be kept indefinitely.

While the information and data resulting from this study may be presented at scientific meetings or published in a scientific journal, your name or other personal information will not be revealed. Some people or groups who receive your health information might not have to follow the same privacy rules. Once your information is shared outside of DUHS, we cannot guarantee that it will remain private. If you decide to share private information with anyone not involved in the study, the federal law designed

to protect your health information privacy may no longer apply to the information you have shared. Other laws may or may not protect sharing of private health information.

WHAT ARE THE COSTS TO YOU?

There will be no costs to you for the research procedures. However, you or your insurance provider will be responsible and billed for all costs related to your routine medical care, including copayments and deductibles. Routine medical care services are those that you would have received for your condition if you were not participating in this research study. Not all services are covered by insurance. Some procedures or scans may require pre-authorization by your insurance plan. We will notify you if we learn that a service is not covered by your insurance plan as part of the pre-authorization process. If it is not covered, you will be responsible for paying for it. The amount of your out-of-pocket expense will depend on your insurance plan. For beneficiaries with Medicare Advantage Plans, traditional Medicare is billed for the routine cost of a research study. You may have more or higher co-pays than with a Medicare Advantage plan. Please discuss the costs of the study with a member of the study team**.** At your request, a Financial Counselor in the clinic may provide you with an estimate of costs for routine services.

WHAT ABOUT COMPENSATION?

You will not be paid for your study participation at Duke. Information about payment your VA participation was provided to you in the VA consent form.

WHAT ABOUT RESEARCH RELATED INJURIES?

Immediate necessary medical care is available at Duke University Medical Center in the event that you are injured as a result of your participation in this research study. However, there is no commitment by Duke University, Duke University Health System, Inc., or your Duke physicians to provide monetary compensation or free medical care to you in the event of a study-related injury.

For questions about the study or research-related injury, contact Dr. Michael at 919-613-5020, or Dr. Young at 919-286-0411, extension 17-7258. .

WHAT ABOUT MY RIGHTS TO DECLINE PARTICIPATION OR WITHDRAW FROM THE STUDY?

You may choose not to be in the study, or, if you agree to be in the study, you may withdraw from the study at any time. If you withdraw from the study, no new data about you will be collected for study purposes other than data needed to keep track of your withdrawal.

Your decision not to participate or to withdraw from the study will not involve any penalty or loss of benefits to which you are entitled and will not affect your access to health care at Duke. If you do decide to withdraw, we ask that you contact Dr. Michael in writing and let him know that you are withdrawing from the study. His mailing address is Duke University Health System, Campus Box 91003, Durham, NC 27708.

Duke University Health System

Consent to Participate in a Research Study: Neuroimaging correlates and feasibility of TMS for smoking cessation in veterans with PTSD

We will tell you about new information that may affect your health, welfare, or willingness to stay in this study.

Your data may be stored and shared for future research without additional informed consent if identifiable private information, such as your name and medical record number, are removed. If your identifying information is removed from your samples or data, we will no longer be able to identify and destroy them.

The use of your data may result in commercial profit. You will not be compensated for the use of your data and samples other than what is described in this consent form.

Your doctor may decide to take you off this study if your condition gets worse, if you have serious side effects, or if your study doctor determines that it is no longer in your best interest to continue. The sponsor or regulatory agencies may stop this study at any time without your consent. Reasons why this might occur include: 1) you have withdrawn from the sister study at the Durham VA, or 2) you are noncompliant with study procedures. If this occurs, you will be notified, and your study doctor will discuss other options with you.

WHOM DO I CALL IF I HAVE QUESTIONS OR PROBLEMS?

For questions about the study or a research-related injury, or if you have problems, concerns, questions or suggestions about the research, contact Dr. Michael at 919-613-5020, or Dr. Young at 919-286-0411, extension 17-7258. You may also contact the study coordinator, Ms. Addison Troutman, at 919-286-0411, extension 17-6702.

For questions about your rights as a research participant, or to discuss problems, concerns or suggestions related to the research, or to obtain information or offer input about the research, contact the Duke University Health System Institutional Review Board (IRB) Office at (919) 668-5111.

Duke University Health System

Consent to Participate in a Research Study: Neuroimaging correlates and feasibility of TMS for smoking cessation in veterans with PTSD

STATEMENT OF CONSENT

"The purpose of this study, procedures to be followed, risks and benefits have been explained to me. I have been allowed to ask questions, and my questions have been answered to my satisfaction. I have been told whom to contact if I have questions, to discuss problems, concerns, or suggestions related to the research, or to obtain information or offer input about the research. I have read this consent form and agree to be in this study, with the understanding that I may withdraw at any time. I have been told that I will be given a signed and dated copy of this consent form."

______________________________ ________ ________
Signature of Subject Date Time

______________________________ ________ ________
Signature of Person Obtaining Consent Date Time
