# Supplementary material for: Multimodal smoking cessation treatment combining repetitive transcranial magnetic stimulation, cognitive behavioral therapy, and nicotine replacement in veterans with posttraumatic stress disorder: A feasibility randomized controlled trial protocol
Source: PLoS One. 2024 Sep 6;19(9):e0291562. doi: 10.1371/journal.pone.0291562 (PMC11379281; doi:10.1371/journal.pone.0291562)
Supplement: S3 Appendix — (PDF) [file pone.0291562.s003.pdf]

Protocol: adult_head_tmssmoke.01

3-Plane Localizer

PATIENT POSITION	
Patient Entry	*Head First*
Patient Position	*Supine*
Coil Configuration	*GE48chHeadPos2;GE48chHeadAnt2*
Plane	*3-PLANE*
Series Description	*3-Plane Localizer*

SCAN TIMING	
TE	*80.0*
Number of Echoes	*1*
TR	*1000.0*
Receiver Bandwidth	*83.33*

IMAGE ENHANCE	
Filter Choice	*None*

USER CVS	
User CV1	*1.00*
User CV42	*1.00*
User CV Mask2	*2048*

MULTI-PHASE	
Seperate Series	*0*
Mask Phase	*0*
Mask Pause	*0*
Preserve	*0*

DIFFUSION	
Recon All Images	*On*
Multi b-values	*1000.0;*
Multi NEX Values	*1.0;*
# Synthetic b-values	*1*
Synthetic b-value	*1000.0;*

CONTRAST	
Contrast Yes/No	*No*

IMAGING PARAMETERS	
Imaging Mode	*2D*
Pulse Sequence	*Spin Echo*
Imaging Options	*Seq, EDR, TRF, Fast, SS*

SCAN RANGE	
FOV	*30.0*
Slice Thickness	*15.0*
Slice Spacing	*5.0*

ACQ TIMING	
Freq	*256*
Phase	*128*
Freq DIR	*Unswap*
# of Acq. Before Pause	*0*
Phase FOV	*1.00*
Auto Shim	*Auto*
Phase Correction	*No*
RF Drive Mode	*Quadrature (CP)*
Excitation Mode	*Selective*

FMRI	
PSD Trigger	*Internal*
View Order	*Bottom/Up*
# of Repetitions REST	*0*
# of Repetitions ACTIVE	*0*

SAT	
Tag Type	*None*

TRICKS	
Pause On/Off	*On*
Auto Subtract	*0*
Auto SCIC	*Off*

OTHERS	
Protocol Notes	*48 channel coil*<br>*GE resp and pulse*<br>*right hand button box*

3-Plane Localizer

Protocol: adult_head_tmssmoke.01

GE HOS FOV28

PATIENT POSITION	
**Patient Entry**	*Head First*
**Patient Position**	*Supine*
**Coil Configuration**	*GE48chHeadPos2;GE48chHeadAnt2*
**Plane**	*AXIAL*
**Series Description**	*GE HOS FOV28*

SCAN TIMING	
**Flip Angle**	*10*
**Number of Echoes**	*2*
**TR**	*Minimum*
**Receiver Bandwidth**	*31.25*

IMAGE ENHANCE	
**Filter Choice**	*None*

USER CVS	
**User CV30**	*1.00*
**User CV42**	*1.00*
**User CV Mask2**	*2048*

MULTI-PHASE	
**Seperate Series**	*0*
**Mask Phase**	*0*
**Mask Pause**	*0*
**Preserve**	*0*

DIFFUSION	
**Recon All Images**	*On*
**Multi b-values**	*1000.0;*
**Multi NEX Values**	*1.0;*
**# Synthetic b-values**	*1*
**Synthetic b-value**	*1000.0;*

CONTRAST	
**Contrast Yes/No**	*No*

IMAGING PARAMETERS	
**Imaging Mode**	*3D*
**Pulse Sequence**	*Gradient Echo*
**Imaging Options**	*EDR, Fast, ARC*
**PSD Name**	*3db0map_hos*
**Phase**	*2.00*

SCAN RANGE	
**FOV**	*28.0*
**Slice Thickness**	*6.6*
**Location per Slab**	*36*
**Overlap Locations**	*0*
**Number of Slices**	*1*

ACQ TIMING	
**Freq**	*64*
**Phase**	*64*
**Freq DIR**	*R/L*
**Phase FOV**	*1.00*
**Auto Shim**	*Off*
**Phase Correction**	*No*
**RF Drive Mode**	*Preset*
**Excitation Mode**	*Selective*

FMRI	
**PSD Trigger**	*Internal*
**View Order**	*Bottom/Up*
**# of Repetitions REST**	*0*
**# of Repetitions ACTIVE**	*0*

SAT	
**Tag Type**	*None*

TRICKS	
**Pause On/Off**	*On*
**Auto Subtract**	*0*
**Auto SCIC**	*Off*

GE HOS FOV28

Protocol: adult_head_tmssmoke.01

GE EPI resting

PATIENT POSITION	
Patient Entry	*Head First*
Patient Position	*Supine*
Coil Configuration	*GE48chHeadAnt2;GE48chHeadPos2*
Plane	*OBLIQUE*
Series Description	*GE EPI resting*

SCAN TIMING	
Flip Angle	*60*
TE	*30.0*
Number of Echoes	*1*
TR	*1500.0*
Number of Shots	*1*

IMAGE ENHANCE	
Filter Choice	*None*

GATING/TRIGGER	
Pause After Navigator Prescan	*0*

FMRI	
Initial State	*Stimulus*
PSD Trigger	*Internal*
View Order	*Bottom/Up*
# of Repetitions REST	*0*
# of Repetitions ACTIVE	*0*

SAT	
Tag Type	*None*
Fat/Water Saturation	*Fat*

TRICKS	
Pause On/Off	*On*
Auto Subtract	*0*
Auto SCIC	*Off*

OTHERS	
Protocol Notes	*** Freq.Dir. should be R/L **<br>phys_record_flag = 1<br>Participant look at cross (+)<br>slices interleaved<br>COIL<br>Coil tab -> click the gear icon in the upper right corner -> select "Manual" -> Make sure all boxes are checked (will need to select the "Neck Posterior" box)<br>*DO NOT click the "Auto" coil option b/c it will reset the coils and the Neck Posterior section will turn back off*<br>**will need to do this for all scans***

IMAGING PARAMETERS	
Imaging Mode	*2D*
Pulse Sequence	*Gradient Echo*
Imaging Options	*EDR, MPh, EPI, ARC, HB*
Phase	*2.00*
HyperBand Slice	*3*

SCAN RANGE	
FOV	*25.6*
Slice Thickness	*2.0*
Slice Spacing	*0.0*
Number of Slices	*66*

ACQ TIMING	
Freq	*128*
Phase	*128*
Freq DIR	*Unswap*
NEX	*1.00*
Phase FOV	*1.00*
Auto Shim	*Off*
Phase Correction	*Yes*
RF Drive Mode	*Preset*
Excitation Mode	*Selective*

USER CVS	
User CV0	*1.00*
User CV7	*1.00*
User CV42	*1.00*
User CV Mask2	*2048*

MULTI-PHASE	
Slice per Location	*320*
Phase Acquisition Order	*Interleaved*
Delay after Acquisition	*Minimum*
Seperate Series	*0*
Delay after Acquisition without AV	*0*
Mask Phase	*0*
Mask Pause	*0*

DIFFUSION	
Recon All Images	*On*
Multi b-values	*1000.0;*
Multi NEX Values	*1.0;*
# Synthetic b-values	*1*
Synthetic b-value	*1000.0;*

CONTRAST	
Contrast Yes/No	*No*

GE EPI resting

Protocol: adult_head_tmssmoke.01

resting reverse polarity

PATIENT POSITION	
Patient Entry	*Head First*
Patient Position	*Supine*
Coil Configuration	*GE48chHeadAnt2;GE48chHeadPos2*
Plane	*OBLIQUE*
Series Description	*resting reverse polarity*

SCAN TIMING	
Flip Angle	*80*
TE	*30.0*
Number of Echoes	*1*
TR	*1500.0*
Number of Shots	*1*

IMAGE ENHANCE	
Filter Choice	*None*

GATING/TRIGGER	
Pause After Navigator Prescan	*0*

FMRI	
Brain Wave Real Time	*2*
Paradigm String	*<<<OBJTASK>**>*>*****
Paradigm UID	*1.2.840.113819.3.116518062708.1256068790.255*
Initial State	*Control*
PSD Trigger	*Internal*
Slice Order	*Interleaved*
View Order	*Bottom/Up*
# of Repetitions REST	*1*
# of Repetitions ACTIVE	*1*
# of Dummy Acquisition	*0*

SAT	
Tag Type	*None*

TRICKS	
Pause On/Off	*On*
Auto Subtract	*0*
Auto SCIC	*Off*

OTHERS	
Protocol Notes	*CV = pepolar = 0*

IMAGING PARAMETERS	
Imaging Mode	*2D*
Pulse Sequence	*Gradient Echo*
Imaging Options	*EDR, EPI, FMRI, ARC, HB*
IDEAL	*1*
Phase	*2.00*
HyperBand Slice	*3*

SCAN RANGE	
FOV	*25.6*
Slice Thickness	*2.0*
Slice Spacing	*0.0*
Number of Slices	*66*

ACQ TIMING	
Freq	*128*
Phase	*128*
Freq DIR	*Swap*
NEX	*1.00*
Phase FOV	*1.00*
Auto Shim	*Auto*
Phase Correction	*Yes*
RF Drive Mode	*Preset*
Excitation Mode	*Selective*

USER CVS	
User CV0	*1.00*
User CV7	*1.00*
User CV42	*1.00*
User CV Mask2	*2048*

MULTI-PHASE	
Slice per Location	*10*
Seperate Series	*0*
Delay after Acquisition without AV	*0*
Mask Phase	*0*
Mask Pause	*0*
Preserve	*0*

DIFFUSION	
Recon All Images	*On*
# Synthetic b-values	*1*
Synthetic b-value	*1000.0;*

CONTRAST	
Contrast Yes/No	*No*

resting reverse polarity

Protocol: adult_head_tmssmoke.01

field map multi echo

PATIENT POSITION	
**Patient Entry**	*Head First*
**Patient Position**	*Supine*
**Coil Configuration**	*GE48chHeadPos2;GE48chHeadAnt2*
**Plane**	*OBLIQUE*
**Series Description**	*field map multi echo*

SCAN TIMING	
**Flip Angle**	*15*
**TE**	*Min Full*
**Number of Echoes**	*3*
**TR**	*100.0*
**Receiver Bandwidth**	*31.25*

IMAGE ENHANCE	
**Filter Choice**	*None*

GATING/TRIGGER	
**Pause After Navigator Prescan**	*0*

FMRI	
**Initial State**	*Control*
**PSD Trigger**	*Internal*
**View Order**	*Bottom/Up*
**# of Repetitions REST**	*0*
**# of Repetitions ACTIVE**	*0*

SAT	
**Tag Type**	*None*

TRICKS	
**Pause On/Off**	*On*
**Auto Subtract**	*0*
**Auto SCIC**	*Off*

OTHERS	
**Protocol Notes**	*-field map CV change rhrcctrl=3 Should write out magnitude, phase*

IMAGING PARAMETERS	
**Imaging Mode**	*2D*
**Pulse Sequence**	*Multi-Echo FGRE*
**Imaging Options**	*FC, Fast, Asset*
**Phase**	*2.00*

SCAN RANGE	
**FOV**	*24.0*
**Slice Thickness**	*3.8*
**Slice Spacing**	*0.0*
**Number of Slices**	*35*

ACQ TIMING	
**Freq**	*64*
**Phase**	*64*
**Freq DIR**	*R/L*
**NEX**	*1.00*
**# of Acq. Before Pause**	*0*
**Phase FOV**	*1.00*
**Auto Shim**	*Off*
**Phase Correction**	*No*
**RF Drive Mode**	*Preset*
**Excitation Mode**	*Selective*

USER CVS	
**User CV16**	*1.00*
**User CV17**	*1.00*
**User CV20**	*1.00*
**User CV42**	*1.00*
**User CV Mask2**	*2048*

MULTI-PHASE	
**Seperate Series**	*0*
**Mask Phase**	*0*
**Mask Pause**	*0*
**Preserve**	*0*

DIFFUSION	
**Recon All Images**	*On*
**# Synthetic b-values**	*1*
**Synthetic b-value**	*1000.0;*

CONTRAST	
**Contrast Yes/No**	*No*

field map multi echo

Protocol: adult_head_tmssmoke.01

3D Ax T1 MPRAGE

PATIENT POSITION	
**Patient Entry**	*Head First*
**Patient Position**	*Supine*
**Coil Configuration**	*GE48chHeadPos2;GE48chHeadAnt2*
**Plane**	*AXIAL*
**Series Description**	*3D Ax T1 MPRAGE*

SCAN TIMING	
**Flip Angle**	*8*
**Number of Echoes**	*1*
**TI**	*900*
**Receiver Bandwidth**	*31.25*
**Recovery Time**	*700*

IMAGE ENHANCE	
**Filter Choice**	*G*

USER CVS	
**User CV6**	*1.00*
**User CV30**	*1.00*
**User CV42**	*1.00*
**User CV Mask2**	*2048*

MULTI-PHASE	
**Seperate Series**	*0*
**Trigger Delay without AV**	*0*
**Mask Phase**	*0*
**Mask Pause**	*0*
**Preserve**	*0*

DIFFUSION	
**Recon All Images**	*On*
**# Synthetic b-values**	*1*
**Synthetic b-value**	*1000.0;*

CONTRAST	
**Contrast Yes/No**	*No*

IMAGING PARAMETERS	
**Imaging Mode**	*3D*
**Pulse Sequence**	*MP-RAGE*
**Imaging Options**	*EDR, Fast, ARC, IrP*
**Phase**	*2.00*
**Slice**	*1.00*

SCAN RANGE	
**FOV**	*25.6*
**Slice Thickness**	*1.0*
**Location per Slab**	*208*
**Overlap Locations**	*0*
**Number of Slices**	*1*

ACQ TIMING	
**Freq**	*256*
**Phase**	*256*
**Freq DIR**	*A/P*
**NEX**	*1.00*
**Phase FOV**	*1.00*
**Auto Shim**	*Auto*
**Phase Correction**	*No*
**RF Drive Mode**	*Preset*
**Excitation Mode**	*Selective*

FMRI	
**PSD Trigger**	*Internal*
**View Order**	*Bottom/Up*
**# of Repetitions REST**	*0*
**# of Repetitions ACTIVE**	*0*

SAT	
**Tag Type**	*None*

TRICKS	
**Pause On/Off**	*On*
**Auto Subtract**	*0*
**Auto SCIC**	*Off*

OTHERS	
**Protocol Notes**	*Include scalp and as much of face as can, include the nose*

3D Ax T1 MPRAGE

Protocol: adult_head_tmssmoke.01

Ax T2

PATIENT POSITION	
Patient Entry	*Head First*
Patient Position	*Supine*
Coil Configuration	*GE48chHeadPos2;GE48chHeadAnt2*
Plane	*AXIAL*
Series Description	*Ax T2*

SCAN TIMING	
Flip Angle	*111*
TE	*138.6*
Number of Echoes	*1*
TR	*11000.0*
Echo Train Length	*21*
Receiver Bandwidth	*41.67*

IMAGE ENHANCE	
Filter Choice	*G*

USER CVS	
User CV12	*1.00*
User CV29	*1.00*
User CV42	*1.00*
User CV Mask2	*3328*

MULTI-PHASE	
Seperate Series	*0*
Mask Phase	*0*
Mask Pause	*0*
Preserve	*0*

DIFFUSION	
Recon All Images	*On*
# Synthetic b-values	*1*
Synthetic b-value	*1000.0;*

CONTRAST	
Contrast Yes/No	*No*

IMAGING PARAMETERS	
Imaging Mode	*2D*
Pulse Sequence	*FSE-XL*
Imaging Options	*EDR, TRF, Fast, ARC*
Phase	*2.00*

SCAN RANGE	
FOV	*25.6*
Slice Thickness	*1.0*
Slice Spacing	*0.0*
Number of Slices	*204*

ACQ TIMING	
Freq	*256*
Phase	*256*
Freq DIR	*A/P*
Fat Shift DIR	*Normal (A)*
NEX	*1.00*
# of Acq. Before Pause	*0*
Phase FOV	*1.00*
Auto Shim	*Off*
Phase Correction	*No*
RF Drive Mode	*Preset*
Excitation Mode	*Selective*

FMRI	
PSD Trigger	*Internal*
View Order	*Bottom/Up*
# of Repetitions REST	*0*
# of Repetitions ACTIVE	*0*

SAT	
Tag Type	*None*

TRICKS	
Pause On/Off	*On*
Auto Subtract	*0*
Auto SCIC	*3*

OTHERS	
Protocol Notes	*Copy T1 Rx*

Ax T2

Protocol: adult_head_tmssmoke.01

task

PATIENT POSITION	
**Patient Entry**	*Head First*
**Patient Position**	*Supine*
**Coil Configuration**	*GE48chHeadAnt2;GE48chHeadPos2*
**Plane**	*OBLIQUE*
**Series Description**	*task*

SCAN TIMING	
**Flip Angle**	*60*
**TE**	*30.0*
**Number of Echoes**	*1*
**TR**	*1500.0*
**Number of Shots**	*1*

IMAGE ENHANCE	
**Filter Choice**	*None*

GATING/TRIGGER	
**Pause After Navigator Prescan**	*0*

FMRI	
**Initial State**	*Stimulus*
**PSD Trigger**	*Internal*
**View Order**	*Bottom/Up*
**# of Repetitions REST**	*0*
**# of Repetitions ACTIVE**	*0*

SAT	
**Tag Type**	*None*
**Fat/Water Saturation**	*Fat*

TRICKS	
**Pause On/Off**	*On*
**Auto Subtract**	*0*
**Auto SCIC**	*Off*

OTHERS	
**Protocol Notes**	*** Freq.Dir. should be R/L **<br>phys_record_flag = 1<br>start task with "t"<br>slices interleaved<br>COIL<br>Coil tab -> click the gear icon in the upper right corner -> select "Manual" -> Make sure all boxes are checked (will need to select the "Neck Posterior" box)<br>*DO NOT click the "Auto" coil option b/c it will reset the coils and the Neck Posterior section will turn back off*<br>**will need to do this for all scans***

IMAGING PARAMETERS	
**Imaging Mode**	*2D*
**Pulse Sequence**	*Gradient Echo*
**Imaging Options**	*EDR, MPh, EPI, ARC, HB*
**Phase**	*2.00*
**HyperBand Slice**	*3*

SCAN RANGE	
**FOV**	*25.6*
**Slice Thickness**	*2.0*
**Slice Spacing**	*0.0*
**Number of Slices**	*66*

ACQ TIMING	
**Freq**	*128*
**Phase**	*128*
**Freq DIR**	*Unswap*
**NEX**	*1.00*
**Phase FOV**	*1.00*
**Auto Shim**	*Off*
**Phase Correction**	*Yes*
**RF Drive Mode**	*Preset*
**Excitation Mode**	*Selective*

USER CVS	
**User CV0**	*1.00*
**User CV7**	*1.00*
**User CV42**	*1.00*
**User CV Mask2**	*2048*

MULTI-PHASE	
**Slice per Location**	*400*
**Phase Acquisition Order**	*Interleaved*
**Delay after Acquisition**	*Minimum*
**Seperate Series**	*0*
**Delay after Acquisition without AV**	*0*
**Mask Phase**	*0*
**Mask Pause**	*0*

DIFFUSION	
**Recon All Images**	*On*
**Multi b-values**	*1000.0;*
**Multi NEX Values**	*1.0;*
**# Synthetic b-values**	*1*
**Synthetic b-value**	*1000.0;*

CONTRAST	
**Contrast Yes/No**	*No*

task

Protocol: adult_head_tmssmoke.01

HCP DTI

PATIENT POSITION	
**Patient Entry**	*Head First*
**Patient Position**	*Supine*
**Coil Configuration**	*GE48chHeadAnt2;GE48chHeadPos2*
**Plane**	*AXIAL*
**Series Description**	*HCP DTI*
**SCAN TIMING**	
**TE**	*Minimum*
**Number of Echoes**	*1*
**TR**	*7471.0*
**Number of Shots**	*1*
**IMAGE ENHANCE**	
**Filter Choice**	*None*
**USER CVS**	
**User CV2**	*1.00*
**User CV5**	*1.00*
**User CV11**	*1090.00*
**TR Min**	*3500.0*
**TR Max**	*17000.0*
**User CV39**	*1.00*
**User CV42**	*1.00*
**User CV Mask2**	*2304*
**MULTI-PHASE**	
**Seperate Series**	*0*
**Mask Phase**	*0*
**Mask Pause**	*0*
**Preserve**	*0*
**DIFFUSION**	
**Optimized TE**	*Yes*
**Diffusion Directions**	*Tensor*
**Number of Diffusion Directions**	*90*
**Number of T2 Images**	*2*
**Dual Spin Echo**	*Off*
**Diffusion Tenser Processing Output**	*No Selection*
**Recon All Images**	*Off*
**Multi b-values**	*3000.0;*
**Multi NEX Values**	*1.0;*
**Real Time Field Adjustment**	*1*
**CONTRAST**	
**Contrast Yes/No**	*No*

IMAGING PARAMETERS	
**Imaging Mode**	*2D*
**Pulse Sequence**	*Spin Echo*
**Imaging Options**	*EDR, Cla, EPI, DIFF, ARC, HB*
**Phase**	*1.00*
**HyperBand Slice**	*3*
**SCAN RANGE**	
**FOV**	*22.0*
**Slice Thickness**	*1.5*
**Slice Spacing**	*0.0*
**Number of Slices**	*92*
**ACQ TIMING**	
**Freq**	*144*
**Phase**	*144*
**Freq DIR**	*R/L*
**Phase FOV**	*1.00*
**Auto Shim**	*On*
**Phase Correction**	*Yes*
**RF Drive Mode**	*Preset*
**Excitation Mode**	*Selective*
**FMRI**	
**PSD Trigger**	*Internal*
**View Order**	*Bottom/Up*
**# of Repetitions REST**	*0*
**# of Repetitions ACTIVE**	*0*
**SAT**	
**Tag Type**	*None*
**Fat/Water Saturation**	*Fat*
**TRICKS**	
**Pause On/Off**	*On*
**Auto Subtract**	*0*
**Auto SCIC**	*Off*
**OTHERS**	
**Protocol Notes**	*Best image quality and performance using 48ch Head Coil<br>multiband 3<br>CV pepolar = 1<br>1.5mm isotropic<br>- b0 (2 sets)<br>2 shells:<br>- b1000 (45directions)<br>- b2000 (45directions)<br>Important Tip:<br>CV11<br>- the Tensor filename must be set to 1090 to activate this protocol*

HCP DTI

Protocol: adult_head_tmssmoke.01

Hyperband DTI reverse polarity

PATIENT POSITION	
**Patient Entry**	*Head First*
**Patient Position**	*Supine*
**Coil Configuration**	*GE48chHeadAnt2;GE48chHeadPos2*
**Plane**	*AXIAL*
**Series Description**	*Hyperband DTI reverse polarity*

SCAN TIMING	
**TE**	*Minimum*
**Number of Echoes**	*1*
**TR**	*5882.0*
**Number of Shots**	*1*

IMAGE ENHANCE	
**Filter Choice**	*None*

USER CVS	
**User CV2**	*1.00*
**User CV5**	*1.00*
**TR Min**	*3500.0*
**TR Max**	*17000.0*
**User CV39**	*1.00*
**User CV42**	*1.00*
**User CV Mask2**	*2304*

MULTI-PHASE	
**Seperate Series**	*0*
**Mask Phase**	*0*
**Mask Pause**	*0*
**Preserve**	*0*

DIFFUSION	
**Optimized TE**	*Yes*
**Diffusion Directions**	*Tensor*
**Number of Diffusion Directions**	*6*
**Number of T2 Images**	*3*
**Dual Spin Echo**	*Off*
**Diffusion Tenser Processing Output**	*No Selection*
**Recon All Images**	*Off*
**Multi b-values**	*2000.0;*
**Multi NEX Values**	*1.0;*
**Real Time Field Adjustment**	*1*

CONTRAST	
**Contrast Yes/No**	*No*

IMAGING PARAMETERS	
**Imaging Mode**	*2D*
**Pulse Sequence**	*Spin Echo*
**Imaging Options**	*EDR, Cla, EPI, DIFF, ARC, HB*
**Phase**	*1.00*
**HyperBand Slice**	*3*

SCAN RANGE	
**FOV**	*22.0*
**Slice Thickness**	*1.5*
**Slice Spacing**	*0.0*
**Number of Slices**	*92*

ACQ TIMING	
**Freq**	*144*
**Phase**	*144*
**Freq DIR**	*R/L*
**Phase FOV**	*1.00*
**Auto Shim**	*On*
**Phase Correction**	*Yes*
**RF Drive Mode**	*Preset*
**Excitation Mode**	*Selective*

FMRI	
**PSD Trigger**	*Internal*
**View Order**	*Bottom/Up*
**# of Repetitions REST**	*0*
**# of Repetitions ACTIVE**	*0*

SAT	
**Tag Type**	*None*
**Fat/Water Saturation**	*Fat*

TRICKS	
**Pause On/Off**	*On*
**Auto Subtract**	*0*
**Auto SCIC**	*Off*

OTHERS	
**Protocol Notes**	*multiband 3*<br>*CV: pepolar = 0*

Hyperband DTI reverse polarity

Protocol: adult_head_tmssmoke.01

GE EPI resting

PATIENT POSITION	
Patient Entry	*Head First*
Patient Position	*Supine*
Coil Configuration	*GE48chHeadAnt2;GE48chHeadPos2*
Plane	*OBLIQUE*
Series Description	*GE EPI resting*

SCAN TIMING	
Flip Angle	*60*
TE	*30.0*
Number of Echoes	*1*
TR	*1500.0*
Number of Shots	*1*

IMAGE ENHANCE	
Filter Choice	*None*

GATING/TRIGGER	
Pause After Navigator Prescan	*0*

FMRI	
Initial State	*Stimulus*
PSD Trigger	*Internal*
View Order	*Bottom/Up*
# of Repetitions REST	*0*
# of Repetitions ACTIVE	*0*

SAT	
Tag Type	*None*
Fat/Water Saturation	*Fat*

TRICKS	
Pause On/Off	*On*
Auto Subtract	*0*
Auto SCIC	*Off*

OTHERS	
Protocol Notes	*** Freq.Dir. should be R/L ** phys_record_flag = 1 Participant look at cross (+) slices interleaved COIL Coil tab -> click the gear icon in the upper right corner -> select "Manual" -> Make sure all boxes are checked (will need to select the "Neck Posterior" box) *DO NOT click the "Auto" coil option b/c it will reset the coils and the Neck Posterior section will turn back off* **will need to do this for all scans***

IMAGING PARAMETERS	
Imaging Mode	*2D*
Pulse Sequence	*Gradient Echo*
Imaging Options	*EDR, MPh, EPI, ARC, HB*
Phase	*2.00*
HyperBand Slice	*3*

SCAN RANGE	
FOV	*25.6*
Slice Thickness	*2.0*
Slice Spacing	*0.0*
Number of Slices	*66*

ACQ TIMING	
Freq	*128*
Phase	*128*
Freq DIR	*Unswap*
NEX	*1.00*
Phase FOV	*1.00*
Auto Shim	*Off*
Phase Correction	*Yes*
RF Drive Mode	*Preset*
Excitation Mode	*Selective*

USER CVS	
User CV0	*1.00*
User CV7	*1.00*
User CV42	*1.00*
User CV Mask2	*2048*

MULTI-PHASE	
Slice per Location	*320*
Phase Acquisition Order	*Interleaved*
Delay after Acquisition	*Minimum*
Seperate Series	*0*
Delay after Acquisition without AV	*0*
Mask Phase	*0*
Mask Pause	*0*

DIFFUSION	
Recon All Images	*On*
# Synthetic b-values	*1*
Synthetic b-value	*1000.0;*

CONTRAST	
Contrast Yes/No	*No*

GE EPI resting
