# Supplementary material for: Multimodal smoking cessation treatment combining repetitive transcranial magnetic stimulation, cognitive behavioral therapy, and nicotine replacement in veterans with posttraumatic stress disorder: A feasibility randomized controlled trial protocol
Source: PLoS One. 2024 Sep 6;19(9):e0291562. doi: 10.1371/journal.pone.0291562 (PMC11379281; doi:10.1371/journal.pone.0291562)
Supplement: S1 File — (DOC) [file pone.0291562.s004.doc]

**PROTOCOL TITLE:** Neuroimaging correlates and feasibility of transcranial magnetic stimulation to improve smoking cessation outcomes in Veterans with posttraumatic stress disorder

**PRINCIPAL INVESTIGATOR:** Jonathan R. Young, MD

**SPONSOR/FUNDING SOURCE:** MIRECC, CSR&D

**Purpose**

The proposed project aims to develop a more effective, neuroscience-informed smoking cessation treatment for Veterans with posttraumatic stress disorder (PTSD). Veterans with PTSD are at increased risk of smoking-related health consequences due to treatment-resistance to current smoking cessation interventions. Thus, more effective treatment options are needed. The US Food and Drug Administration recently cleared a form of non-invasive brain stimulation, repetitive transcranial magnetic stimulation (rTMS), as a smoking cessation treatment for adults. However, it is unclear whether rTMS would be effective for Veterans, especially those with PTSD. The proposed research aims to advance VA healthcare by studying rTMS in Veterans with PTSD who desire to quit smoking and to personalize rTMS therapy with the use of functional magnetic resonance imaging (fMRI). This line of research may not only develop a more effective treatment for smoking cessation for Veterans with PTSD but may also lead to improved treatment options for Veterans with other substance use disorders and co-occurring mental illness.

**Background and Significance**

There has been increasing understanding of the neurobiological mechanisms underlying tobacco use disorder (TUD), as well as the brain regions involved in successful smoking cessation during a quit attempt. This improved understanding of the pathophysiology at the neural systems-level suggests the possibility of utilizing circuit-modulating neurotechnologies to target pathological activity and thereby alleviate symptoms. Noninvasive brain stimulation (NIBS) modalities such as repetitive transcranial magnetic stimulation (rTMS) are able to safely modify neural activity within specific brain regions, as well as induce changes in network cortical function, and ultimately, behavior (Hallet, 2000, 2007; Daskalakis et al, 2006; Wagner et al, 2007). Neuromodulation procedures such as rTMS may provide a safe and effective way to non-invasively target abnormally functioning neurocircuitry found in substance use disorders, including tobacco use disorder. In fact, as of August 2020, the US Food and Drug Administration (FDA) has granted clearance for the use of deep TMS for smoking cessation in adults (Globe Newswire, 2020). This approval builds on growing evidence that rTMS is an effective device-based intervention that could serve as an alternative or adjunct to current evidence-based treatments for smoking cessation, including pharmacotherapy and psychotherapy (reviewed in Hauer et al, 2019). Despite this, existing research has been done in civilian populations and there is a lack of published studies using rTMS for smoking cessation in Veterans, particularly those with PTSD. A review of currently enrolling clinical trials in the US shows that none investigating rTMS for smoking cessation are recruiting individuals with PTSD, and most exclude individuals with comorbid psychiatric illness (National Library of Medicine, NLM). Furthermore, few prior studies in this space used neuronavigation to accurately target personalized, neuroimaging-based cortical regions.

While the ideal brain stimulation target is unknown, rTMS applied over the superior frontal gyrus (SFG) demonstrated differential effects on cravings, depending on the cue presented: high-frequency 10-Hz TMS applied to SFG increased cravings to smoking cues, yet decreased cravings to neutral cues (Rose et al, 2011). In another study, 20-Hz rTMS was applied sequentially over the left DLPFC and the medial SFG over 10 days, which resulted in a significant reduction in nicotine cravings as well as resting brain activity as measured by the functional magnetic resonance imaging (fMRI) measures, cerebral blood flow and brain entropy (Chang et al, 2018). In the largest published trial (n=115), rTMS was applied to lateral prefrontal cortex and insula bilaterally using a BrainsWay H4 deep TMS (dTMS) coil following presentation of smoking cues over 13 sessions in a population of heavy smokers with failed previous treatments, high- and low-frequency stimulation resulted in significantly reduced cigarette consumption with an abstinence rate of 44% at the end of treatment and an estimated 33% at 6-month follow-up (Dinur-Klein et al, 2014). This work led to an unpublished multicenter RCT using the H4 dTMS coil in chronic, heavy smokers (n=262 enrolled, n=168 completers), which demonstrated after 3 weeks of treatments plus 3 weeks of follow-up a 28.4% continuous quit rate in the active group compared to 11.7% in the sham group (p=0.0063). Based on this study, the FDA granted 510(k) clearance in August 2020 for dTMS as an aid in short-term smoking cessation in adults (Globe Newswire, 2020). Despite this exciting and important development, this trial excluded patients with recent psychiatric illness and those using NRT, smoking cessation drugs or behavioral therapy (National Library of Medicine, NLM). The currently proposed study will help fill this gap by assessing rTMS as a smoking cessation treatment in a special population that was excluded from prior trials (Veterans with PTSD) while offering NRT and psychotherapy.

The activity of the insula and connected neural network have been found to be important to the success of smoking cessation attempts. A study evaluating cigarette use after brain damage demonstrated that damage to the insula was significantly more likely to result in smoking cessation when compared to damage that spared the insula (Naqvi et al, 2007). Others have demonstrated the role of the insula in the subjective, interoceptive awareness of drug craving, which contributes to motivation to use tobacco and other substances (Naqvi & Bechara, 2010). As part of a larger brain network, resting-state functional connectivity and enhanced cue reactivity of the insula have implicated this brain region in tobacco smoking relapse vulnerability during smoking cessation treatment (Addicott et al, 2015; Janes et al, 2017).

Using resting-state functional MRI (rs-fMRI), greater functional connectivity between the posterior insula and postcentral gyrus was found to correlate with abstinence during a quit attempt (Addicott et al, 2015). This finding suggests that strengthening this circuit could be therapeutic and improve abstinence rates. Through a combination of rs-fMRI and rTMS, pathological circuits may be optimally modulated and accelerate the translation of these techniques into treatment (Fox et al, 2012). Thus, in a follow-up, proof-of-principle study conducted by mentor, Dr. Appelbaum, and colleagues, it was shown that rTMS was able to non-invasively modulate activity of the insula in a healthy civilian population (Addicott et al, 2019). In this study, 1- or 10-Hz rTMS was applied daily for 5 days to a personalized target on the postcentral gyrus most functionally connected to the insula. rs-fMRI with the insula as a seed region was measured before and after the treatment course. Interestingly, despite the hypotheses that 10-Hz rTMS would increase and 1-Hz rTMS would decrease connectivity, both stimulation parameters increased functional connectivity of the cortical stimulation target and the insula (Addicott et al, 2019). These results support the potential for clinical impact of rs-fMRI-based rTMS for smoking cessation. Indeed, this approach is currently being pursued in the treatment of major depressive disorder (MDD). An open-label trial of the Stanford Accelerated Intelligent Neuromodulation Therapy (SAINT) for treatment-resistant depression (TRD) recently demonstrated feasibility and clinical utility of functional connectivity MRI as a method for precision targeting of the left DLPFC, resulting in 86.4% of subjects achieving remission on intent-to-treat analysis (Cole et al, 2020).

**Gaps in Knowledge:** Despite advances in neuromodulation treatment modalities, it is unknown whether these preliminary findings of therapeutic fMRI-based rTMS generalize to Veterans who smoke tobacco. There is insufficient data on whether such an intervention would make a clinically significant impact, particularly for those with comorbid psychiatric illness such as PTSD. In addition, it is unknown whether Veterans would find this combined neuroimaging-brain stimulation approach acceptable and if the multiple steps required for precision rTMS for smoking cessation is feasible.

Thus, the primary goal of this project is to evaluate the feasibility and acceptability of rTMS applied to a personalized, functional connectivity-based target. Integrated into smoking cessation treatment as an adjunctive intervention to standard-of-care, evidence-based, cognitive behavioral counseling for smoking cessation and pharmacotherapy, this proposal also seeks to determine whether Veterans with PTSD are willing to enroll in and complete the research activities of a pilot feasibility study in which functional connectivity of the insula guides individualized, neuronavigated, rTMS therapy. The study will include two phases: 1) an open label single group feasibility trial, and 2) a parallel design, double-blinded, sham-controlled, randomized trial comparing active- vs sham- intermittent theta burst rTMS. Primary outcomes for phase one will be recruitment, retention, acceptability, and feasibility, and primary outcomes for phase two will be recruitment and retention. Phase two will also include an efficacy aim evaluating 7-day point prevalence at the end-of-treatment, and 3-months post-quit date using previously established methods of self-reported abstinence and bioverification. We hypothesize that rs-fMRI-guided rTMS will be acceptable and feasible by Veterans with PTSD and that we will be able to successfully recruit and retain subjects for the pilot study. Proposed is an open-label, single-group pilot feasibility study in 14 Veterans with tobacco use disorder and comorbid PTSD.

**Veteran Health Relevance**: In the United States, an estimated 44.5 million adults smoke cigarettes, causing death or disability in approximately half of this population (Mokdad et al, 2004). When compared to other major causes of mortality, there are more tobacco-related deaths than those related to AIDS, illicit substance use, alcohol consumption, motor vehicle accidents, suicides and homicides combined (Centers for Disease Control and Prevention, 2002). Individuals with mental illness are disproportionately affected by the medical burden of tobacco use (Sokal et al, 2004). In one American study, smokers with psychiatric disorders accounted for more than 200,000 of the 520,000-annual tobacco-attributed deaths and are dying an average 25 years prematurely (Colton & Manderscheid, 2006). In addition to significant public health consequences of tobacco smoking, the economic impact is more than $96 billion per year in medical expenses, as well as $97 billion per year in lost productivity (Centers for Disease Control and Prevention, 2008).

The proposed work is of vital importance to the health of Veterans given the high prevalence of both PTSD and tobacco use disorder in this population. PTSD has been diagnosed in 11% of Veterans receiving care from the VA, which has a financial impact of over $250 million annually (Greenberg & Hoff, 2016). PTSD is the most prevalent psychiatric disorder among Afghanistan/Iraq era Veterans, with rates approaching 32% in this population (Veterans Health Administration, DoVA & Epidemiology Program, 2017). In Veterans with PTSD, the risk of tobacco use disorder is tripled (Smith et a., 2016). Furthermore, the rates of smoking in patients with psychiatric disorders has been increasing (Cook et al, 2014). Unfortunately, individuals with PTSD have the lowest rates of successful smoking cessation efforts when compared to those with other psychiatric disorders (Fu et al, 2007), thus leading to higher risk of morbidity and mortality.

The desire to quit smoking among Veterans is not lacking. Approximately 60-70% of all Veterans who smoke tobacco express interest in smoking cessation (Miller et al, 2001). A strong dose-response relationship between treatment intensity and cessation rates does exist (Fiore et., 2000). The most efficacious strategies found in VA smoking cessation clinics combine multiple formats of cognitive-behavioral interventions, including self-help materials, group therapy, and telephone counseling, resulting in a 23% abstinence rate (Fiore et al, 2000). These rates worsen in the context of comorbid psychiatric conditions, with specialty-based care smoking cessation efforts negatively affected by limited access and poor adherence (Jonk et al, 2005). Clinic attendance rates at specialty smoking cessation clinics are approximately 13-14% for all Veterans, and those with PTSD even less likely to follow up (McFall et al, 2010). Unfortunately, among Veterans with PTSD who do present to smoking cessation clinics, the efficacy of interventions is lower (McFall et al, 2010). This was demonstrated in the largest smoking cessation trial in Veterans with PTSD, with a biochemically verified (bio-verified; exhaled CO < 8 ppm or urine cotinine < 100ng/mL) cessation rate of only 4.5% at 12-months (McFall et al, 2010). These statistics underscore the urgency to consider alternative interventions for smoking cessation in this clinical population, including neuromodulaiton modalities such as rTMS.

In addition to the physical benefits of quitting smoking, there are enhanced mental health clinical outcomes associated with tobacco-cessation treatments. Quitting smoking has even been associated with reductions in PTSD symptoms (Prochaska, 2010). In a smoking cessation study of 577 Veterans, abstinence from tobacco at both 2- and 6-month follow-up points was associated with reduced overall psychiatric symptoms (Krebs et al, 2018). This has been replicated in a larger meta-analysis of 26 tobacco intervention studies that found that smoking cessation was significantly associated with decreased anxiety, depression, stress, as well as improvements in overall mood and quality of life (Taylor et al, 2014). Smoking cessation has even been associated with a 25% increased likelihood of sobriety from alcohol and illicit drugs (Prochaska et al, 2004). Overall, these findings confirm the significant clinical benefits of successful smoking cessation interventions, especially for Veterans with comorbid psychiatric disorders.

Demonstrating feasibility of rTMS for smoking cessation in a Veteran population diagnosed with PTSD will provide critical insight into whether this now-FDA cleared therapeutic modality may play a role in the clinical management of tobacco use disorder for Veterans, especially in populations with high levels of treatment-resistance. If the scientific and training aims are achieved, RCT efficacy studies will be indicated, and the candidate will be in an ideal position to carry out this work in the CDA-2. The long-term goal of this work is to ultimately advance clinical practice by introducing a novel, safe, and effective intervention that will benefit Veteran psychiatric and medical well-being.

**Design**

**Phase 1 Research Design and Methods**: To determine the viability of rTMS as a smoking cessation intervention in a special clinical population of Veterans with comorbid PTSD, we propose to conduct an open-label feasibility study to evaluate the ability to provide and acceptability of rs-fMRI-guided rTMS in Veterans with PTSD who smoke who are receiving standard-of-care for smoking cessation at Durham VA. In addition, we propose to evaluate the ability to recruit and retain Veterans in the feasibility study. A single-arm, open-label feasibility study was chosen based on the specific aims, small sample size limiting statistical analyses, and practical considerations of the CDA-1 including 2-year duration, lack of research funding, and explicitly prohibited RCT design in the CDA-1 funding announcement.

We hypothesize that fMRI-guided rTMS will be safe, feasible, acceptable by Veterans, and we will be able to effectively recruit and retain eligible subjects in the study. We expect to meet the primary endpoints of the proposed project, which will determine successful completion of the study intervention and justification to continue this line of research. Specifically, this will be defined as recruitment of 18 subjects, 14 subjects completing at least 90% of all study procedures (~80% retention rate), and an acceptability rate of ≥ 7/10 on a 10-point scale. We also hypothesize that pre-treatment rs-fMRI will provide a cortical target in the right postcentral gyrus that is functionally connected with the insula and accessible by neuronavigated rTMS, therefore establishing the feasibility of the protocol. Exploratory outcomes will include a change in fMRI resting-state functional connectivity between the cortical rTMS target and the insula, therefore demonstrating target engagement. While not the focus of the current study, we will also monitor clinical outcomes to demonstrate if these measures can be collected, including nicotine dependence, cravings, withdrawal, and abstinence (biochemically verified by exhaled carbon monoxide ≤ 6 ppm), psychiatric symptoms related to PTSD and depression, and alcohol use.

Overall Design (see Figure 1): Fourteen Veterans who are heavy smokers (>10 cigarettes daily) and diagnosed with PTSD will complete 5 daily rs-fMRI-guided rTMS treatments the week prior to their quit date, in addition to receiving standard-of-care, evidence-based treatment for smoking cessation 5 weekly smoking cessation counseling sessions plus NRT that includes at least one nicotine rescue method (e.g., patch, gum, inhaler). Subjects will undergo pre-rTMS fMRI and post-rTMS fMRI scans. Outcome measures will be assessed at baseline before (pre-rTMS), after the treatment course on the quit date, and at 2-week follow-up. All rTMS and FMRI procedures will occur at Duke University Medical Center (DUMC). Participants will sign a Duke consent form and HIPAA authorization for those procedures.

Primary outcomes to be evaluated will be recruitment and retention of subjects (AIM 1), and feasibility and acceptability of study procedures (AIM 2). We will determine whether we have met our primary end points by defining recruitment as successfully enrolling 18 subjects, retention as having 14 subjects complete at least 90% of all study procedures (~20% attrition rate), feasibility as completing the study intervention including rs-fMRI-based neuronavigation and rTMS treatments as well as follow-up measures, and acceptability as an average score of ≥ 7/10 on a 10-point acceptability scale.

Secondary/exploratory outcomes to be evaluated pre- and post-rTMS will be functional connectivity changes on rs-fMRI scans; levels of nicotine dependence using the Fagerström Test for Nicotine Dependence (FTND); nicotine cravings using the Brief Questionnaire of Smoking Urges and a 100-point Urge to Smoke Scale; nicotine withdrawal on the Minnesota Nicotine Withdrawal Scale-Revised (MNWS-R); abstinence rates verified using exhaled carbon monoxide (CO); PTSD symptoms as measured by the PTSD Checklist 5 (PCL-5), depressive symptoms as measured by the BDI-II; alcohol use as measured by the Alcohol Use Disorder Alcohol Use Disorders Identification Test Consumption Screening Tool (AUDIT-C); and illicit substance use as measured by the Drug Abuse Screening Test (DAST). We will also evaluate subjective distress during TMS procedures using the Subjective Units of Distress Scale (SUDS).

Figure 1. Phase 1 Overall Design

**Quit Date**

**Week: 1 2**  **3 4 5**

Nicotine Replacement Therapy (NRT)


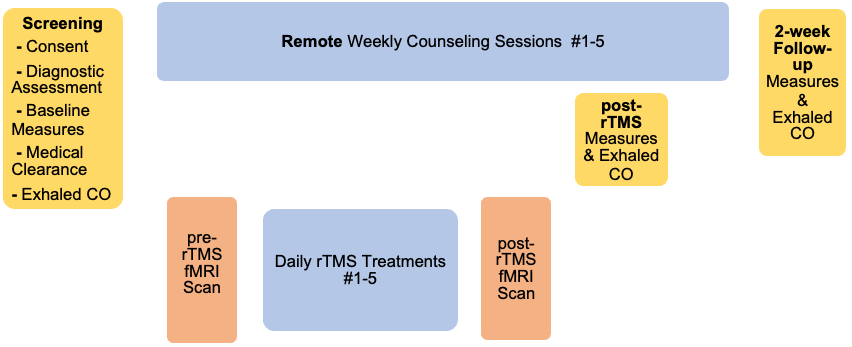


VA:

Duke:

Secondary/exploratory outcomes to be evaluated pre- and post-rTMS will be functional connectivity changes on rs-fMRI scans; levels of nicotine dependence using the Fagerström Test for Nicotine Dependence (FTND); nicotine cravings using the Brief Questionnaire of Smoking Urges and a 100-point Urge to Smoke Scale; nicotine withdrawal on the Minnesota Nicotine Withdrawal Scale-Revised (MNWS-R); abstinence rates verified using exhaled carbon monoxide (CO); PTSD symptoms as measured by the PTSD Checklist 5 (PCL-5), depressive symptoms as measured by the BDI-II; alcohol use as measured by the Alcohol Use Disorder Alcohol Use Disorders Identification Test Consumption Screening Tool (AUDIT-C); and illicit substance use as measured by the Drug Abuse Screening Test (DAST). We will also evaluate subjective distress during TMS procedures using the Subjective Units of Distress Scale (SUDS).

**Phase 1 Study Procedures**

Screening Visit: During the COVID-19 pandemic, participants will provide informed consent and HIPAA authorization via mail via DocuSign where available. Subjects will be screened for safety issues by completing the TMS Adult Safety Screen (TASS; Keel et al, 2001), the Duke MRI Safety Screening form, and the Ohio State University (OSU) TBI Identification Method-Short Form. Subjects will also complete self-report measures and receive a clinician-administered psychiatric interview (see Table 1). Study inclusion and exclusion criteria will be used to determine eligibility. If eligible, the rest of the study visits will be scheduled based on availability of the participant as well as Duke procedures (fMRI scanner, rTMS device).

Medical Screening: Medical clearance for subjects to be enrolled will be made by study physician and mentor, Scott Moore, M.D., Ph.D., and applicant, Jonathan Young, M.D., using the study eligibility criteria, as well as negative results on the TMS Adult Safety Screen (TASS), MRI Safety and OSU TBI screening forms.

Biochemical Verification of Abstinence: Participants will provide exhaled breath carbon monoxide (CO) levels at baseline, the quit date and the 2-week follow-up. Breath CO levels are an effective method for monitoring for cessation, and it has an additional positive impact on shaping and inducing smoking abstinence in the first week of a quit attempt (Benowitz, Hukkanen et al, 2009). If participating remotely, participants will be provided with CO monitors to use at these time points and will perform the CO reading during a telehealth visit in which they can show the results via video; otherwise, this will be performed in-person at a VA facility. We will use a standardized cutoff value of ≤ 6 ppm (Beckham et al, 2018). This cut-off value has previously been identified as optimal (Woodward & Tunstall-Pedoe, 1992), with good sensitivity and specificity among outpatients (Middleton & Morice, 2000).

Exploratory smoking outcomes will include point prevalence abstinence based on subject self-report and confirmed using remote-monitored exhaled CO level at the quit date and the 2-week follow-up assessment. Additional measures will be daily number of cigarettes smoked, subjective levels of withdrawal, craving, and smoking urges.

DUMC Study Procedures: All MRI procedures will occur at DUMC. Participants will undergo an initial functional MRI scan (pre-rTMS fMRI) to locate a personalized target site for rTMS All rTMS treatments will occur at DUMC. MagVenture’s MAGPRO X100 will be used for rTMS procedures. A cue provocation procedure to induce nicotine cravings will be conducted prior to rTMS treatments using a video of smoking-related images as well as instructions for handling of a cigarette and lighter. Following the last rTMS session, a second fMRI scan (post-rTMS fMRI) will be conducted at least 1 hour and up to 24 hours later.

Adaptations due to COVID-19: All screening procedures, psychotherapy sessions, and non-fMRI follow-up assessments may be conducted remotely using VA-approved secure platforms (e.g., VA Video Connect, VA WebEx,MyHealthEVet). As fMRI and rTMS procedures will occur at Duke, the study team will follow DUMC policies for participant COVID screening.

Measures: The following measures will be obtained to ensure subject eligibility, determine whether subjects are willing to complete these questionnaires, and to characterize the sample for the CDA-2 pilot. Where possible, participants will complete coded study measures via Qualtrics using their own electronic devices (e.g., smart phones, computers, tablets). If a participant does not have a device available, they will be allowed to complete the measures by mail or in person. All data will be stored at VA Qualtrics, which is FedRAMP approved for collection and storage of VA PHI and VA sensitive information.

Subject Sociodemographic and Clinical Background Variables: Participants will complete a background sociodemographic and military service measure at screening to characterize the subject sample.

Diagnostic Assessment and Psychiatric Measures: A thorough evaluation for psychiatric conditions will be completed using the Structured Clinical Interview for DSM-5 (SCID-5; First et al, 2015). To ensure subjects meet study criteria for a PTSD diagnosis, the Clinician Administered PTSD Scale for DSM-5 (CAPS-5), a semi-structured interview considered to be the “gold standard” for PTSD diagnostic assessment (Weathers et al, 2015), will be used. PTSD symptoms will be monitored using the past-month and past-week versions of the PCL-5, a 20-item self-report measure successfully used in Veterans for monitoring symptom change with treatment (Blevins et al, 2015; Bovin et al, 2015). The Inventory of Depressive Symptomatology – Self Report (IDS-SR; Rush, Carmody, & Reimitz. 2006) will be used to monitor depressive symptoms, illicit substance use will be monitored using past-year and past month versions of the DAST (Bohn et al, 1991), and alcohol use will be tracked using the 3-item AUDIT-C (Bush et al, 1998).

Tobacco-Related Measures: Tobacco-related measures will include the FTND (Heatherton et al, 1991), the MNWS-R (Hughes, 2012), the Brief Questionnaire on Smoking Urges, and a 100-point scale on Smoking Urges. These scales will characterize levels of dependence on nicotine include number of cigarettes smoked daily, withdrawal, and cravings. We will also monitor tobacco use with a calendar for timeline follow back.

rTMS-Related Measures: To assess Veterans’ perceived credibility and expectancy of TMS procedures, the Credibility/Expectancy Questionnaire (Devilly & Borkovec, 2000) will be administered at baseline and post-rTMS/quit date. To determine Veterans’ acceptability of the intervention, we will measure a patient satisfaction questionnaire tailored to this study’s treatment aims, administered at the post-rTMS/quit date and 2-week follow-up assessments. To determine Veterans’ tolerability of the intervention, a brief TMS side effect rating scale will be administered at the post-rTMS/quit date and 2-week follow-up assessments. We will also assess subjective distress using the Subjective Units of Distress Scale (SUDS).

Table 1. Measures Administered in Phase 1 at Major Study Time Points

| **Measure** | **Time Point** | | |
| --- | --- | --- | --- |
|  | **Baseline** | **Post-rTMS / Quit Date** | **2-week Follow-Up** |
| Sociodemographic and Military Background | X |  |  |
| **Diagnostic Assessment and Psychiatric Measures** | | | |
| SCID-5 | X |  |  |
| CAPS-5 (past month) | X |  |  |
| PCL-5 (past month) | X | X | X |
| BDI-II | X | X | X |
| AUDIT-C | X | X | X |
| DAST (past year) | X |  |  |
| DAST (past month) | X | X | X |
| **Tobacco-Related Measures** | | | |
| FTND | X | X | X |
| MNWS-R | X | X | X |
| Brief Questionnaire on Smoking Urges | X | X | X |
| 100-point Urge to Smoke Scale | X | X | X |
| Remote-monitored exhaled CO |  | X | X |
| **rTMS-Related Measures** | | | |
| Credibility/Expectancy Questionnaire | X | X |  |
| TMS Side Effects Scale |  | X | X |
| Satisfaction Questionnaire |  | X | X |
| Structured Clinical Interview for DSM-5 = SCID-5; Clinician Administered PTSD Scale for DSM-5 = CAPS-5; PTSD Checklist for DSM-5 = PCL-5; Beck Depression Inventory-II = BDI-II; Alcohol Use Disorders Identification Test Consumption Screening Tool = AUDIT-C; Fagerström Test of Nicotine Dependence = FTND; Minnesota Nicotine Withdrawal Scale-Revised = MNWS-RS; DAST = Drug Abuse Screening Tool | | | |

Data for diagnostic and follow-up measures will be collected by study staff in Dr. Beckham’s Traumatic Stress and Health Research Laboratory. Our group has trained diagnostic interviewers for our studies using training videos, observation of live interviews, and monthly meetings to prevent rater drift. Inter-rater reliability across previous interviewers has been excellent (kappa = .96). Neuroimaging data will be collected by MRI technologists in BIAC. rTMS procedures will be administered by the candidate with the assistance of Dr. Appelbaum and his staff in BSRC.

**Phase 1 Selection of Subjects**

The primary goal of the current study is to evaluate the feasibility, acceptability, recruitment and retention of Veterans with posttraumatic stress disorder (PTSD) for a treatment for smoking cessation that includes functional magnetic resonance imaging (fMRI)-guided repetitive transcranial magnetic stimulation (rTMS). For this feasibility study, we plan to screen 26 potential subjects, recruit/enroll 18 subjects (based on 8 not meeting eligibility criteria) and retain 14 subjects who complete at least 90% of all study procedures (based on a ~20% attrition rate). All human subjects in this project will be adult male and female Veterans. Potential participants will be recruited from the Durham Veterans Affairs Health Care System (DVAHCS). Study procedures will occur at both Duke University Medical Center (DUMC) and DVAHCS. Study inclusion and exclusion criteria are outlined below.

Phase 1 Inclusion/Exclusion Criteria:

| Subjects must meet **all** inclusion criteria: | Subjects must not have **any** exclusion criteria: |
| --- | --- |
| | - Is a US Veteran - Is between the ages of 18 and 75 (see Rossi et al., 2021) - Smokes an average of ≥10 cigarettes per day for the past 6 months, with carbon monoxide (CO) level > 6 ppm - Is willing to attempt smoking cessation - Meets DSM-5 criteria for current PTSD diagnosis - Speaks, reads and writes English - Currently an outpatient at DVAHCS - Is willing and able to participate in rTMS intervention, MRI and all required study visits - Is willing to sign a Duke consent for those portions of the study that occur at Duke. | | --- | | - Has previously received TMS - Has a current substance use disorder other than tobacco - Has a contraindication to TMS or MRI   - Personal or family history of a seizures or epilepsy   - History of neurological condition that increases the risk of seizures including stroke or transient ischemic attack, cerebral aneurysm, or severe traumatic brain injury from a penetrating head injury, loss of consciousness > 20 minutes at time of traumatic injury, requiring an anticonvulsant medication for seizures, and/or found to have encephalomalacia on baseline MRI   - Structural brain lesion, or prior brain surgery   - Ferromagnetic metal in head (including shrapnel)   - Implanted devices that may be affected by MRI or TMS (pacemaker, medication pump, cochlear implant, implanted deep brain stimulator)   - Is pregnant (to be determined at Duke) - Is unable to complete study procedures |

**Phase 2 Research Design and Methods**: The proposed study is a two-arm, parallel design, double-blinded, sham-controlled, randomized trial comparing active- vs sham- rTMS neuronavigated to a region on the post-central gyrus functionally connected to the insula in addition to evidence-based CBT for smoking cessation and NRT.

Overall Design: Fifty eligible participants will be randomized to one of 2 conditions: active-rTMS + CBT + NRT (n=25) or sham-rTMS + CBT + NRT (n=25). The full randomization sequence will be generated *a priori*. Participants will receive a baseline MRI scan, 5 daily neuronavigated active or sham rTMS treatments the week prior to their quit date, followed by a post-treatment MRI scan. In order to be able to use the fMRI results to inform placement of the TMS coils, there may be up to a two-week lag between the first MRI and the first TMS treatment session. All participants will receive 5 sessions of CBT for smoking cessation in which session 3 is designated as the quit date. All participants will also receive prescriptions of nicotine replacement in the form of 21 mg, 14 mg, and/or 7 mg patches and 2-4 mg nicotine gum or lozenges starting on the quit date and continuing for up to 90 days at individually tapered doses. All rTMS and MRI procedures will occur at Duke University Medical Center (DUMC). Participants will sign a Duke consent form and HIPAA authorization for those procedures.

We hypothesize that neuronavigated rTMS combined with CBT and NRT will be a safe, feasible, and effective method for smoking cessation in our study population. We anticipate being able to effectively recruit and retain eligible subjects in the study. Our primary feasibility endpoint will help determine successful completion of the study intervention and justification to continue this line of research. Recruitment will target enrolling 50 subjects, with a goal of 40 or more subjects completing at least 90% of all study procedures (>80% retention rate). We hypothesize that active- as compared to sham-rTMS will result in greater 7-day point prevalence abstinence rates (biochemically verified by exhaled carbon monoxide < 5 ppm) at end-of-treatment, and 3-month follow-up time points. We also hypothesize that active-rTMS will result in an increase in functional connectivity between the cortical rTMS target and the insula, therefore demonstrating target engagement. We also anticipate network-level differences in rsFC between active- vs sham-rTMS groups. We will also monitor levels of nicotine dependence, cravings, withdrawal, prolonged abstinence, psychiatric symptoms related to PTSD and depression, and alcohol use.

**The primary outcome** to be evaluated in the feasibility aim will be recruitment and retention of subjects, with a target enrollment of 50 subjects and 80% retention rate (i.e., 40 completers). For the efficacy aim, we will estimate the effect size by evaluating 7-day point prevalence at the end-of-treatment, and 3-months post-quit date using previously established methods of self-reported abstinence and bioverification. For the target engagement aim, we will evaluate functional connectivity between the rTMS target on sensorimotor cortex and right posterior insula.

**Secondary/exploratory outcomes** will be network-level changes on rsFC using the right posterior insula as a region-of-interest; levels of nicotine dependence using the Fagerström Test for Nicotine Dependence (FTND); nicotine cravings using the Brief Questionnaire of Smoking Urges; nicotine withdrawal on the Minnesota Nicotine Withdrawal Scale-Revised (MNWS-R); PTSD symptoms as measured by the PCL-5, depressive symptoms as measured by the BDI-II; alcohol use as measured by the alcohol use disorders identification test-consumption (AUDIT-C) screening tool; and illicit substance use as measured by the drug abuse screening test (DAST).

**Phase 2 Study Procedures**

Screening Visit: These study procedures are similar to Phase 1 of the project. Once considered eligible to participate in the study, participants will be randomized 1:1 to receive either active rTMS or sham rTMS. Participants will remain blinded to study condition throughout the study. Dr. Young and Dr. Moore will also remain blinded to study condition.

Medical Screening: These study procedures are similar to Phase 1 of the project. However, in lieu of one screening session, we will divide the screen tasks between two visits. Diagnostic interviews will be completed at the first visit, and questionnaires and CO readings will be completed at the second.

Biochemical Verification of Abstinence: Participants will provide exhaled breath carbon monoxide (CO) levels at baseline, quit date, end-of-treatment, and at 3-month follow-up visit. We will use a standardized cutoff value of < 5 ppm. In addition, at the post-treatment and 3-month follow-up visit, urinary cotinine will also be collected for more comprehensive bioverification for those participants who are no longer using NRT. Urine samples collected will be sent to LabCorp for analysis.

DUMC Study Procedures: MRI procedures are identical to Phase 1 MRI procedures. MagVenture’s MagPro X100 will be used for rTMS procedures. rTMS parameters will be updated from Phase 1 to Phase 2 due to advances in the field and Phase 1 data. Phase 1 will be 5 daily rTMS sessions of 1 Hz 960 pulses for 16 minutes each. Phase 2 will be 5 days of rTMS treatments twice daily using patterned rTMS, intermittent theta burst stimulation (iTBS; 3-pulse 50-Hz bursts at 5-Hz for 2-s trains, with trains every 10 s) 1800 pulses at 90% rMT for 9 minutes each separated by at least 50 minutes. Active- or sham-rTMS will be determined by utilizing a subject-specific code recognized by the TMS device as active or sham, which then instructs staff as to which side of the TMS coil to use. The Magventure B65 A/P coil has identical sides, but the computer will know which side to use based on each participant code. A cue provocation procedure to induce nicotine cravings will be conducted prior to rTMS treatments using a video of smoking-related images as well as instructions for handling of a cigarette and lighter. Following the last rTMS session, a second fMRI scan (post-rTMS fMRI) will be conducted at least 1 hour and up to 24 hours later.

Additional Study Interventions: Smoking cessation counseling and nicotine replacement therapies are identical to those offered in Phase 1. Participants will be offered NRT for at least 60 days.

Measures: The following measures will be obtained to ensure subject eligibility, determine whether subjects are willing to complete these questionnaires, and to characterize the sample for the CDA-2 pilot. Where possible, participants will complete coded study measures via Qualtrics using their own electronic devices (e.g., smart phones, computers, tablets). If a participant does not have a device available, they will be allowed to complete the measures by mail or in person. All data will be stored at VA Qualtrics, which is FedRAMP approved for collection and storage of VA PHI and VA sensitive information.

Subject Sociodemographic and Clinical Background Variables: Participants will complete a background sociodemographic and military service measure at screening to characterize the subject sample.

Diagnostic Assessment and Psychiatric Measures: A thorough evaluation for psychiatric conditions will be completed using the Structured Clinical Interview for DSM-5 (SCID-5; First et al, 2015). To ensure subjects meet study criteria for a PTSD diagnosis, the Clinician Administered PTSD Scale for DSM-5 (CAPS-5), a semi-structured interview considered to be the “gold standard” for PTSD diagnostic assessment (Weathers et al, 2015), will be used. PTSD symptoms will be monitored using the past-month version of the PCL-5, a 20-item self-report measure successfully used in Veterans for monitoring symptom change with treatment (Blevins et al, 2015; Bovin et al, 2015). The BDI-II will be used to monitor depressive symptoms (Beck et al, 1996), illicit substance use will be monitored using past-year and past month versions of the DAST (Bohn et al, 1991), and alcohol use will be tracked using the 3-item AUDIT-C (Bush et al, 1998).

Tobacco-Related Measures: Tobacco-related measures will include the FTND (Heatherton et al, 1991), the MNWS-R (Hughes, 2012), the Brief Questionnaire on Smoking Urges, and a 100-point scale on Smoking Urges. These scales will characterize levels of dependence on nicotine include number of cigarettes smoked daily, withdrawal, and cravings. At baseline, participants will participate in a cue provocation procedure to induce (and measure) nicotine cravings. The procedure includes watching a brief video with smoking-related images, with instructions for the participant to handle a cigarette and lighter. At three time points, we will ask participants about their motivation to quit smoking and confidence to stop smoking long term. The question regarding motivation to quit will only be asked of those participants who are still smoking at posttreatment and follow-up. We will also monitor tobacco use with a calendar for timeline follow back.

rTMS-Related Measures: To assess Veterans’ perceived credibility and expectancy of TMS procedures, the Credibility/Expectancy Questionnaire (Devilly & Borkovec, 2000) will be administered at baseline and post-rTMS/quit date. To determine Veterans’ acceptability of the intervention, we will measure a patient satisfaction questionnaire tailored to this study’s treatment aims, administered at the post-rTMS/quit date and 2-week follow-up assessments. To determine Veterans’ tolerability of the intervention, a brief TMS side effect rating scale will be administered at the post-rTMS/quit date and 2-week follow-up assessments. We will also assess subjective distress using the Subjective Units of Distress Scale (SUDS).

Table 2. Measures Administered in Phase 2 at Major Study Time Points

| **Measure** | **Time Point** | | | |
| --- | --- | --- | --- | --- |
|  | **Baseline** | **Quit Date** | **End-of-treatment** | **3-Month**  **Follow-up** |
| Sociodemographic and Military Background | X |  |  |  |
| MRI Safety Screening | X |  |  |  |
| TMS Safety Screening | X |  |  |  |
| OSU-TBI | X |  |  |  |
| ***Diagnostic Assessment and Psychiatric Measures*** | | | | |
| SCID-5 | X |  |  |  |
| CAPS-5 (past month) | X |  |  |  |
| PCL-5 (past month) | X | X | X | X |
| IDS-SR | X | X | X | X |
| AUDIT-C | X | X | X | X |
| DAST (past year) | X |  |  |  |
| DAST (past month) | X | X | X | X |
| ***Tobacco-Related Measures and Bioverification*** | | | | |
| FTND | X | X | X | X |
| MNWS-R | X | X | X | X |
| Brief Questionnaire on Smoking Urges | X | X | X | X |
| *Exhaled CO* | X | X | X | X |
| Motivation to quit and confidence to stay quit items | X |  |  | X |
| Cue Provocation/Cravings Measure | X |  |  |  |
| *Urinary Cotinine* |  |  |  | X |
| ***rTMS-Related Measures*** | | | | |
| Credibility/Expectancy Questionnaire | X | X |  |  |
| TMS Side Effects Scale |  | X | X | X |
| Acceptability Questionnaire |  | X | X | X |
| Treatment Allocation Guess |  | X | X | X |
| MRI = magnetic resonance imaging; TMS = transcranial magnetic stimulation; OSU-TBI = Ohio State University-Traumatic Brain Injury Identification Method-Short Form; Structured Clinical Interview for DSM-5 = SCID-5; Clinician Administered PTSD Scale for DSM-5 = CAPS-5; PTSD Checklist for DSM-5 = PCL-5; Inventory of Depressive Symptomatology-Self Report = IDS-SR; Alcohol Use Disorders Identification Test Consumption Screening Tool = AUDIT-C; Fagerström Test of Nicotine Dependence = FTND; Minnesota Nicotine Withdrawal Scale-Revised = MNWS-RS; DAST = Drug Abuse Screening Tool | | | | |

**Phase 2 Selection of Subjects**

We estimate that approximately 120 Veterans will be consented and screened, with 50 being enrolled and randomized to a study condition, and a final sample of 40 participants who complete all study procedures. However, given that the subsequent full RCT will use intent-to-treat analysis, we will include all 50 participants in descriptive statistics and primary analyses. Study inclusion and exclusion criteria are outlined below.

Phase 2 Inclusion/Exclusion Criteria:

| Subjects must meet **all** inclusion criteria: | Subjects must not have **any** exclusion criteria: |
| --- | --- |
| | - Is a US Veteran - Meets DSM-5 criteria for tobacco use disorder - Is between the ages of 18 and 75 - Smokes an average of ≥10 cigarettes per day for the past 6 months, with carbon monoxide (CO) level > 6 ppm - Is willing to attempt smoking cessation - Meets DSM-5 criteria for current PTSD diagnosis - Speaks, reads and writes English - Is willing to sign a Duke consent for those portions of the study that occur at Duke - Has been stable on psychotropic medications for at least three months | | --- | | - Has had a substance use disorder other than tobacco in the preceding 3 months - Has a history of myocardial infarction in the past 6 months or has another contraindication to NRT - Has a contraindication to TMS or MRI   - Personal or family history of a seizures or epilepsy   - History of neurological condition that increases the risk of seizures including stroke or transient ischemic attack, cerebral aneurysm, or severe traumatic brain injury from a penetrating head injury, loss of consciousness > 20 minutes at time of traumatic injury, requiring an anticonvulsant medication for seizures, and/or found to have encephalomalacia on baseline MRI   - Structural brain lesion, or prior brain surgery   - Ferromagnetic metal in head (including shrapnel)   - Implanted devices that may be affected by MRI or TMS (pacemaker, medication pump, cochlear implant, implanted deep brain stimulator)   - Is pregnant (to be determined at Duke) - Is unable to complete study procedures - Is currently prescribed bupropion and/or varenicline - Uses other forms of nicotine such as cigars, pipes, chewing tobacco, or vaping - Is unable to provide informed consent due to a major neurocognitive disorder or other reason - Meets criteria for a primary psychotic disorder or current manic episode - Is currently imprisoned or psychiatrically hospitalized - Has previously received rTMS |

**Phase 1 and Phase 2 Subject Recruitment**

Based on group’s previous study that had 14 subjects per group (Addicott et al, 2019) and a meta-analysis of online rTMS studies published by mentor, Dr. Appelbaum, demonstrating an average sample size of 11 in similar studies (Beynel et al, 2019), we plan to have 14 subjects complete all the procedures in the proposed feasibility study. Based on Dr. Beckham’s previous research in the study population (Carpenter et al, 2015), we anticipate a similar attrition rate of approximately 20%. Therefore, we expect to enroll 18 subjects in order to have 14 complete the majority of study procedures. In order to enroll 18 eligible study participants, we estimate that we will need to consent and screen approximately 26 potential participants. The Durham VA Medical Center (DVAMC) will be the recruitment site from which Veterans will be identified. Participants will be recruited primarily from among outpatients at the DVAHCS PTSD clinic, Operation Enduring Freedom/Operation Iraqi Freedom/Operation New Dawn (OEF/OIF/OND) clinic, Women’s Health clinic, and Mental Health clinic, as well as IRB-approved letters inviting people to participate in the study. As further detailed below, we will use proactive recruitment methods to identity (via VA computerized medical records), contact (via introductory letter, toll free opt-out telephone number, and telephone calls), to recruit and consent eligible Veterans. These proactive methods are associated with much higher recruitment rates than those seen in efficacy trials that most typically rely on a reactive recruitment approach (Velicer et al, 2006). In addition, Dr. Dedert will refer subjects who either screen out or who have not achieved smoking abstinence at the end of their participation in his study. In addition, the Traumatic Stress and Health Research Laboratory has a contact database (see IRB #1080), which contains the contact information of participants in previous studies who have indicated (in writing) a desire to be contacted about other research projects in our lab. Any participant who has expressed interest in learning about participating in studies in our lab will be contacted by phone to ask about participation.

We will use VA Data Access Request Tracker (DART) requests to identify additional Veterans with PTSD and tobacco use disorders. We have used similar procedures to successfully recruit Veterans with PTSD in the past. Any potential participant who is identified via a DART request will be sent a recruitment letter and/or an encrypted email (using Azure) that provides basic information about the study. The letter will inform Veterans that they will be contacted by phone in the coming days regarding their interest in participating in the study. In the letter or email, potential participants will be given an “opt-out” number to call in order to decline participation and/or further contact regarding participation. One to two weeks after the mailing, Veterans who have not called to decline participation will be called by a study staff member to request their participation in the research study.

**Phase 1 and Phase 2 Consent Process**

If any participant contacts or is contacted by the study coordinator regarding participation, a telephone script will be used to inform them about the study and do a preliminary determination of eligibility. If after the telephone screening a participant is considered potentially eligible for participation, he/she/they will be sent the study ICF and HIPAA authorization via DocSign, and will be scheduled for a review of the consent documents by phone or video. They will be asked to mail the documents to the study coordinator and will be scheduled for an initial screening session upon receipt. Participants will also be given a copy of the signed informed consent form and phone numbers to call if they have additional questions about the consent form or the research, if they have any problems during the study, or if they have questions about participating in research studies in general. No study procedures will begin until informed consent has been obtained.

**Phase 1 and Phase 2 Study Interventions**

Smoking Cessation Counseling: Dr. Beckham’s laboratory will provide trained therapists to administer five weekly evidence-based smoking cessation counseling sessions. Cognitive behavioral therapy (CBT) for smoking cessation will be provided by a therapist who has been trained in CBT administration. All therapists are rigorously trained and receive ongoing supervision. The counseling sessions will be done via video or telephone.

Smoking Cessation Pharmacotherapy: All eligible participants will be prescribed NRT of at least one nicotine rescue method (e.g., patch, gum, inhaler) at the medical screening evaluation and continued as needed during the duration of the study. NRT will be prescribed by the study physician and mentor, Scott Moore, M.D., Ph.D. Dr. Moore will write the prescriptions and work with participants' primary care physician or psychiatrist to discuss any contraindications. The applicant, Jonathan Young, M.D., will serve as a back-up prescriber in the event of Dr. Moore’s absence. If no contact from the primary care physician can be made, the participants' health information will be evaluated by the study physician, who will determine medical clearance to participate in the trial. Neither varenicline nor bupropion will be prescribed due to the potential for increased risk of seizures during rTMS (FDA, 2015; Little and Ebbert, 2016).

Because the study procedures are not targeting PTSD symptoms, participants will be informed during the informed consent process that they will not be asked to stop any ongoing PTSD treatments, nor will they be asked to refrain from beginning new treatments.

**Phase 1 and 2 Costs and/or Payments to Subjects**

Phase 1: Subjects will receive payments ($100/session) for all assessment procedures completed including baseline screening and two follow-up assessments. We will also pay participants $100 each for receipt of their DUMC study data at VA. This reimbursement strategy is consistent with our prior work (CSR&D Merit CX001486). Subjects will receive a total of $500 if all procedures are completed and data are received, Participants will receive free smoking cessation counseling and a 5-day course of rTMS treatment. Participants will receive NRT via a telehealth clinic and may be charged co-pays as indicated by their eligibility status.

Phase 2: Participants will be paid $100 each of the following visits: screening, quit date, end of treatment, and 3-month follow-up. Participants will also be paid a total of $200 each for receipt of their Duke fMRI data at VA. Participants can receive up to a total of $600 for phase 2 of the study.

**Phase 1 and 2 Data and Safety Monitoring, Including Adverse Events**

Phase 1 of the study will utilize a data and safety monitor to enhance participant protection. The individual responsible for data and safety monitoring will be Tracey C. Holsinger, MD, a board-certified, staff psychiatrist at the Durham VAMC with over 25 years of clinical and research experience in brain stimulation techniques. Dr. Holsinger will review adverse events and monitor the safety of participants as well as the quality and completeness of the accrued study data during regularly scheduled meetings. The data monitor will review data quarterly but will also be consulted at any time in the event of study-related, unanticipated and/or serious adverse events. The data monitor performs the critical function of monitoring study adverse events and determining whether a favorable risk-benefit ratio justifies continuation of a given study. The data monitor plays a complimentary role to the Durham VA Institutional Review Board (IRB) and the Duke University IRB, which focus mainly on the prospective review of study protocols (although the IRB may also be involved in halting a study in the face of serious, reportable adverse events. Any concerns raised by the data monitor will be discussed with the PI and mentoring team.

For phase 2 of the study, data monitoring will be completed by a CSR&D-assigned Data Monitoring Committee (DMC). The study team will submit reports to the DMC annually, or as required by the DMC.

The PI will meet at least weekly with study personnel to discuss enrollment, participation, and any adverse events or unanticipated problems. Regular meetings between investigators and the project manager will allow for ongoing progress reports, including the number of participants currently involved in each study, attrition rates, and scheduled data collection from participants, as well as notification and review of any AEs. Safety monitoring for AEs will be conducted in real time by the PI and/or project manager. The following information about adverse events will be collected: 1) the onset and resolution of the AE, 2) an assessment of the severity or intensity (use existing grading scales whenever possible), 3) an assessment of the relationship of the event to the study (definitely, probably, possibly or not related), and 4) action taken (e.g., none, referral to physician, start or increase concomitant medication). The PI will determine the severity of the event, will assign attribution to the event, and will monitor the event until its resolution. Any adverse events will be reported to the DVAHCS and DUMC IRBs in accordance with their Human Research Protection Program’s Standards of Practice. All research projects conducted at DVAHCS and DUMC are required to have yearly IRB review, including a safety review. Additionally, any changes to the project between review periods must be approved by the appropriate IRB prior to fielding.

Based on the study team’s long-term clinical and research experience with patients with high-risk psychiatric disorders such as PTSD, depression, and anxiety disorders, we have developed IRB-approved standards of practice for psychiatric emergencies. These standards have been used for several years. It is not unexpected that participants will experience increased distress associated with the diagnostic clinical interviews. Our extensive clinical and research experience suggest that there is no serious risk in these patients associated with assessment and interview procedures as proposed. Over the past several years, several hundred patients with psychiatric diagnoses have participated in our research team’s clinical trials at DVAHCS and DUMC, and there have been only rare occurrences of serious adverse events (i.e., hospitalization only) due to temporary psychiatric symptom increases.

**Phase 1 and 2 Risk/Benefit Assessment**

**Potential Risks**

Overall, the risks to subjects are low in light of the potential benefits to them for participating in the study, including quitting smoking. Participants may receive satisfaction from contributing to the progress of scientific research.

Study-related serious adverse events are not expected but will be closely monitored and will be reported in accordance with VA, DUMC, and FDA policy.

Study Assessments: Regarding completion of study measures and structured interviews, there is a risk of discomfort or distress in answering questions, especially questions related to traumatic experiences. However, distress and discomfort related to these study activities are usually temporary and well-tolerated. Previous studies in our laboratory that have included trauma- and PTSD-related questionnaires, interviews, and treatment have had no greater than expected adverse events related to study participation. Every effort will be made to make the environment comfortable and supportive during assessment procedures. Risks also include discomfort related to quitting smoking. Quitting smoking will cause nicotine withdrawal that may lead to headaches, nausea, irritability, weight gain, difficulty concentrating, poor sleep, increased appetite, anxious or depressed mood, and craving for cigarettes. There are risks associated with the use of NRT. Minimal risks associated with wearing a nicotine patch include skin irritation, dizziness, lightheadedness, increased heart rate or blood pressure, nausea or vomiting. Inherent to any research, there is also a potential risk associated with the loss of confidentiality of study data.

Please note, risks associated with study procedures that occur at DUMC are described in the Duke IRB protocol and informed consent document.

**Phase 1 and 2 Protection Against Risk**

Participants are informed clearly during the initial informed consent process that the study is voluntary, and that they may refuse to answer any items that they do not wish to answer on the questionnaires and interviews. They are also informed that they are free to decline participation in any procedure and can withdraw from the study at any time.

Our study team has an established IRB-approved standard of practice (SOP) for the evaluation of risk of suicide and homicide. All study staff members are trained in use of the psychiatric emergencies SOP by a Ph.D. or Masters Degree-level clinician with years of experience in working with persons with psychiatric disorders. The SOP includes a thorough risk assessment including evaluation of risk factors and protective factors associated with both suicide and homicide. Also included in the policy are differential recommendations for action based on determinations of low, moderate, or high risk. Any staff member conducting an interview in which moderate or high risk is determined will contact a senior staff person with clinical expertise in risk assessment, including the PI, co-investigator(s), and/or DVAHCS’s Psychiatric Emergency Clinic or the Emergency Department.

At several time points throughout the study, participants are reminded that they are asked to inform the study therapist, study coordinator, or study PI if they experience a psychiatric emergency such as homicidal or suicidal ideation. Finally, participants are provided the telephone number of study staff who they can call in the case of psychiatric emergencies, including an after-hours contact number.

To ensure confidentiality, all records will be identified by the participant’s identification number, not by name. All raw hard copy data will be kept in a locked file cabinet in a locked room on DVAHCS property. Data files will be stored in a limited access folder on a secure server to which only study staff members have access.

Only the study PI, co-investigators, study physician, project coordinator, and research assistants will have access to private health information (PHI) as part of the study. All staff with access to PHI will be required to complete Privacy Awareness and HIPAA Awareness educational modules. Data are managed and protected in accordance with the research data and safety standards of practice at DVAHCS and DUMC. All project staff will complete educational units required by the DVAHCS and DUMC IRBs, which include information security, privacy awareness, and human subjects’ protection.

**Phase 1 and 2 Withdrawal of Participants**

Participants may voluntarily withdraw from participation at any time. The study staff may withdraw a participant one or more of the following reasons: failure to follow the instructions of the study staff; inability to complete the study requirements; or inability to reach participant by telephone after multiple attempts.

**Phase 1 and 2 Study Communication**

For any participant who indicates that they are willing to receive study communications via email, we will use Azure to send secure communications to them re: appointment scheduling, missed appointments, etc. Content from the study’s IRB-approved letters will be included in the emails. Participants will also be provided with a dedicated study phone number to call with any questions or concerns.

**Phase 1 and 2 Data Collected at Duke**

All study data that are collected at Duke, including MRI results, will be transferred to a VA secured server (see storage locations listed below) using a VA-owned encrypted thumb drive.

**Data Analysis and Statistical Considerations**

**Phase 1:** Based on requirements of the CDA-1 that a controlled intervention trial is not allowed, feasibility is the primary focus of the proposed study. Without a control group, neither a power analysis nor effect size calculation are indicated. However, primary endpoints have been operationalized to allow for quantitative assessment. Specifically, we will determine if we meet successful recruitment as enrolling 18 subjects, retention as 14 subjects completing 90% of study procedures (which corresponds to ~20% attrition rate), and acceptability as ≥ 7/10 on 10-point satisfaction scale.

We will conduct an exploratory analysis of the secondary outcome data in order for the candidate to gain experience with analysis of neuroimaging and psychometric data, to prepare for a pilot efficacy trial in the CDA-2, and for descriptive purposes for the CDA-2 application. Given the small number of subjects and large number of variables, we will focus on the correlation between changes on RSFC pre- and post-rTMS and change in the number of cigarettes smoked in the past 24 hours, since we do not expect a change in psychiatric symptoms. We will calculate confidence intervals around the effect size to gain an idea of the variability in the subsequent trial.

**Phase 2:** Feasibility endpoints have been operationalized to allow for quantitative assessment. Specifically, we will determine if we meet successful recruitment by enrolling 50 subjects and retention with 40 subjects completing 90% of study procedures (which corresponds to a 20% attrition rate). We will determine if Veterans find the intervention acceptable based an average score of ≥ 7/10 on a 10-point satisfaction scale.

Primary smoking outcome will be 7-day point prevalence abstinence based on subject self-report and confirmed using exhaled CO level at each follow-up assessment (i.e., end-of-treatment, 3-months post-quit date), with abstinence rates averaged over all assessments to take advantage of all available data and adjust for within-subjects clustering of data.^115^ This definition of abstinence is consistent with those previously used in studies of smoking cessation in Veterans.^28^ Non-abstinence will be defined as self-reported smoking (or other tobacco use) for 7 consecutive days or at least once a week for 2 consecutive weeks. Secondary outcomes will include daily number of cigarettes smoked, subjective levels of withdrawal, craving, and urges to smoke, 7-day point prevalence at 3-months post-quit date, as well as smoking relapse, defined as smoking 5 or more cigarettes per day for 3 consecutive days. We will evaluate 7-day point prevalence smoking abstinence by calculating a dichotomous outcome variable (abstinent or not abstinent) to determine the proportion of participants with bioverified abstinence at each assessment from quit date through 3 months follow-up. Due to the relatively small sample size, we will describe the rates of smoking abstinence by calculating the proportion of participants who are confirmed to be abstinent. We will calculate confidence intervals around the effect size to determine the level of variability expected in the subsequent full RCT.

Participants will be considered abstinent if they meet certain criteria, which we have used in prior work.^97^ For participants taking NRT at the time of follow-up, they will be considered abstinent if they self-report prolonged abstinence and provide a CO reading < 5 ppm. For those not taking NRT, abstinence will be based on: a) self-reported prolonged abstinence, b) absence of any biochemical samples indicating smoking (CO ≥ 5 ppm or cotinine ≥ 6 ng/mL), and c) presence of at least one biochemical sample indicating abstinence (CO < 5 ppm and/or cotinine < 6 ng/mL).

Comparisons of continuous demographic and clinical data will be completed with linear regressions. Comparisons of dichotomous demographic and clinical data will use generalized linear models using a binomial distribution. Regarding 7-day point prevalence smoking abstinence outcomes at end-of-treatment, and 3-months; odds ratios and 95% confidence intervals will be computed from estimates obtained in generalized linear models. A longitudinal analysis using generalized linear models will also be conducted on abstinence across end-of-treatment, and 3-month time periods with predictors of treatment arm, time in months (standardized and centered), and the treatment arm-by-time interaction. All missing smoking data will be imputed as non-abstinent. All analyses will be conducted in R version 4.0.4.^116^

We will also complete analysis of neuroimaging data to determine target engagement. Specifically, we will focus on the correlation between changes on rsFC pre- and post-rTMS and change in the number of cigarettes smoked in the past 24 hours. We will also use a data-driven approach by employing group independent component analysis (ICA) to compare functional network connectivity (FNC) between active- and sham-rTMS groups including FC between rTMS target and right posterior insula.^117^

For the neuroimaging data, movement and scan artifacts will be detected using Artifact Detection Tools^118^ and included in the rsFC analyses as covariates of no interest. Group-level analyses will be conducted in SPM12. rsFC analyses will be confined to a bilateral insula regional of interest (ROI) mask defined from the Automated Anatomical Labeling (AAL) atlas with a significance threshold of p < .001 uncorrected, cluster extent > 10 voxels. Exploratory whole brain analyses will also be conducted with a significance threshold of p < .001 uncorrected, cluster extent > 50 voxels.

In the event that software required for analysis are not available through VINCI, deidentified data will be moved to Duke University Medical Center for analysis. Data will be emailed.

**Phase 1 and 2 Privacy, Confidentiality, and Information Security**

1. **Lists of Data Reviewed and/or Collected for Screening/Recruitment and Conduction of Study:**

The Personal Health Information that will be obtained, used, and/or shared for this study includes:

| **Identifier(s)** | **Source(s) of Health Information** |
| --- | --- |
| Names | Medical history & physical exam information |
| All geographic subdivisions smaller than a State, including street address, city, county, precinct, and zip code. Describe: Participants addresses will be collected for initial recruitment letters; participants may be sent study correspondence by mail. | Photographs, videotapes, audiotapes, or digital or other images |
| All elements of dates (except year) for dates directly related to an individual, including birth date, admission date, discharge date, visit or treatment dates, etc.; and all ages over 89, Describe: Dates of visits will be collected. | Biologic specimens (e.g., blood, tissue, urine, saliva). Describe: |
| Telephone numbers | Progress notes |
| Fax numbers | Diagnostic / Laboratory test results |
| Electronic mail addresses | Operative reports |
| Social Security Numbers | Imaging (x-ray, CT, MRI, etc.) |
| Medical record numbers | Discharge summaries |
| Health plan beneficiary numbers | Survey / Questionnaire responses |
| Account numbers | Billing records |
| Certificate and/or license numbers | HIV testing or infection records |
| Vehicle identifiers and serial numbers, including license plate numbers | Sickle cell anemia information |
| Device identifiers and serial numbers | Alcoholism or alcohol use information |
| Web Universal Resource Locators (URLs) | Drug abuse information |
| Internet Protocol (IP) address numbers | Mental health (not psychotherapy) notes |
| Biometric identifiers, including finger & voice prints | Psychological test results |
| Full-face photographic images and any comparable images | Genetic testing |
| Any other unique identifying number, characteristic, or code, describe :  **Note: This is not the unique code assigned to otherwise de-identified health information for re-identification purposes.* | Other, describe: |

1. **Data and/or Specimen Acquisition:**

Data for this study will be collected through (*check all that apply*):

Prospective data and/or specimen collection obtained from participants. Provide description of processes: Questionnaires, neuroimaging (fMRI), exhaled air (CO monitoring).

Retrospective data collection and/or specimens obtained from medical chart review/data access. Describe how data will be obtained (e.g., fileman, CDW, etc.): The study team will collect and use diagnostic data and contact information from the Corporate Data Warehouse for the purposes of recruitment. .

Retrospective data collection and/or specimens obtained from an IRB-approved data and/or specimen repository. Indicate the repository source including name, VA location, and IRB number:      .

*Note: for data and/or specimens obtained from a VA approved data repository, a Data Use Agreement (DUA) must be executed prior to obtaining data and/or specimens. See VHA Handbook 1200.12 for further information.*

1. **Level of Data:**

The following level(s) of data will be acquired/maintained for this study (*check all that apply*):

Identifiable—Data contains direct identifiers.

Coded—Data linked to a specific by a code rather than a direct identifier for re-identification purposes. Only someone possessing the key to the code can link the data to a particular participant.

De-Identified (all 18 HIPAA identifiers removed

Verified Statistically

OR

Verified by Absence or Removal of 18 HIPAA identifiers

Limited Data Set

Other: Describe:

1. **Location of Data and/or Specimens, and Data Retention Plan:**

A. Data and/or Specimen Location: Data will be stored electronically in \\v06.med.va.gov\dur\Research\Nicotine Research\Study Information\Study Logbooks\rTMS CDA and \\v06.med.va.gov\dur\Research\Nicotine Research\Study Information\Study Databases\rTMS CDA. Data that will be stored electronically include name, address, phone number, social security number, amount of study payment earned, and date of visits (in Study Logbooks location). The study logbook will contain the key connecting PHI and the study identification number. Paper records of data may include study consent form and HIPAA authorization (identified), questionnaire responses, and interview notes (coded). These will be stored in a locked filing cabinet in VA-leased space at 3022 Croasdaile Drive, Durham, NC. or on the main Durham VA campus in Building 1, C10006 or Building 8, Room 206. Data will also be stored within VA Qualtrics, which is FedRAMP approved for collection and storage of PHI and sensitive information, and has an Authority to Operate (ATO) within VA. Data will be moved from Qualtrics \\VHADURFPC02B\groups1\Nicotine Research\Study Information\Study Databases\FOCUS Merit Review for permanent storage. Urine samples will be sent to LabCorp for cotinine analysis.

Data will be also be placed at the VA Informatics and Computing Interface (VINCI; <http://vaww.vinci.med.va.gov/vincicentral/VINCIWorkspace.aspx>). The VA Informatics and Computing Infrastructure is a partnership between the VA Office of Information Technology and the Veterans’ Health Administration Office of Research and Development. Researchers and operations staff can use VINCI to access data and statistical analysis tools in a virtual working environment through a certified VHA network computer using the VA Intranet or Virtual Private Network (VPN).

B. Data Retention Plan

Research records will be maintained and destroyed according to the National Archives and Records Administration, Records Schedule Number: DAA-0015-2015-0004. Records destruction, when authorized, will be accomplished using the then current requirements for the secure disposal of paper and electronic records. Currently, destruction of research records (see DAA-0015-2015-0004, section 7.6 “Research Investigator Files” for materials included in research records) is scheduled for 6 years after the cut-off (the cut-off is the completion of the research project) and may be retained longer if required by other federal agencies. Records will not be destroyed without pre-notification to the facility records manager. .

Other data retention plan, describe:

1. **Data Access and Data Recipients**: At VA, only study staff members will have access to VA data. Participants who enroll in the VA study will be asked to sign a Duke consent form which contains elements of HIPAA authorization for Duke. Participants will first sign the VA consent form and HIPAA authorization, and will then sign the Duke consent form. Staff members consenting participants are joint VA/Duke employees.

All VA research personnel who have access to VHA records are instructed, in accordance with VA policy, on the requirements of Federal privacy and information laws and regulations, VA regulations and policies, and VHA policy. All study personnel who are VA employees working within the VA system have fulfilled all required HIPAA and other VA security and privacy policy training requirements and have agreed to follow guidelines pertaining to the protection of patient data. All research staff sign VA Rules of Behavior, and all study staff are up-to-date with VHA Privacy Policy Training and the VA Office of Cyber and Information Security Awareness Training Course. The data security and privacy procedures summarized in that course include logging off or locking the computer when walking away from it; no sharing of access codes, verify codes or passwords; not allowing anyone else to use the computer under one’s password; and disposing of sensitive information using VA-approved methods (e.g., shredder bins).

Access to study data will be removed for all study personnel when they are no longer part of the research team.

In the event that statistical analysis software is not available in VINCI, a de-identified data set will be sent via email to Duke University, where software is available.

1. **Data and/or Specimen Transportation and/or Transmission for all data and/or specimens involved in the study:**

1. Data and/or specimens will not be transported or transmitted outside of Durham VAMC environment.
2. Data and/or specimens will be transported BETWEEN sites that are under the auspices of the Durham VA Medical Center.
3. Data and/or specimens will be transmitted to other VA sites using the following method(s):
4. **Data**

Data are de-identified and thus will be sent via unencrypted e-mail or unencrypted disk (encryption is optional).

Data are coded or contain identifiers and thus will be sent <.

Other, describe:

1. **Specimens**

Specimens are de-identified and thus will be sent via standard carrier (tracking is optional).

Specimens are coded or contain identifiers and thus will be sent via VA-authorized carrier with tracking.

Other, describe:

1. Data and/or specimens will be transported to non-VA/VHA sites (e.g., academic affiliates, laboratories, etc.) using the following method(s):
2. **Data**

Data are de-identified and thus will be sent via unencrypted e-mail or unencrypted CD.

Data are coded or contain identifiers and thus will be sent via

Data are coded or identified and will be sent via the Safe Access File Exchange (SAFE) at <https://safe.amrdec.army.mil/safe/>. SAFE is a secure method of exchanging files <2GB to and from individuals with a valid .gov, .mil, .com, or .edu email address. <insert information including collaborator name.>

Data are coded or identified and will be uploaded to sponsor website using electronic case report form (eCRF) <insert information including sponsor name and URL and the encryption the site uses.>

Other, describe: Participants who enroll in the VA study will be asked to sign a Duke consent form which contains elements of HIPAA authorization for Duke. Participants will first sign the VA consent form and HIPAA authorization, and will then sign the Duke consent form. Staff members consenting participants are joint VA/Duke employees.

1. **Specimens**

Specimens are de-identified and thus will be sent via standard carrier (tracking is optional) or will be hand-delivered by research study personnel. Specify method of delivery:

Specimens are coded and thus will be sent via VA-approved carrier with tracking or will be hand-delivered by research study personnel. Specify method of delivery: Urine specimens will be sent to LabCorp for analysis. LabCorp will pick up samples directly from 3022 Croasdaile Drive, where study visits will occur.

In accordance with the HIPAA and the Privacy Act, for any coded or identifiable data or specimens released from the Durham VAMC (with the exception of Limited Data Sets), an Accounting of Disclosure (AOD) will be maintained (e.g., in a database or spreadsheet) that includes the participant’s name, date of the disclosure, description of the nature of the Individually Identifiable Information (III) disclosed, purpose of each disclosure, and the name and address of the person/agency to whom the disclosure was made.

**C.**  Local DVAMC memorandum “Authorization to Use, Process, Store, or Transmit VA Sensitive Information Outside VA Owned or Managed Facilities” has been pre-filled out for each study team member who may transport the data and/or specimens off-site. This (these) forms are included with the IRB materials. <

**D.**  Containers (e.g., briefcase, bin) are labeled with the following notice (label placed on the outside of container) in accordance with VHA Directive 6609:

NOTICE!!!

Access to these records is limited to: AUTHORIZED PERSONS ONLY.

Information may not be disclosed from this file unless permitted by all applicable legal authorities, which may include the Privacy Act; 38 U.S.C. §§ 5701, 5705, 7332; the Health Insurance Portability and Accountability Act; and regulations implementing those provisions, at 38 C.F.R. §§ 1.460 – 1.599 and 45 C.F.R. Parts 160 and 164. Anyone who discloses information in violation of the above provisions may subject to civil and criminal penalties.

1. We will communicate with veterans enrolled as participants in this research study through MyHealtheVet.
2. **Risk Mitigation Strategies:**

Data are fully de-identified (stripped of HIPAA 18 and study ID/code) before being shared outside of Durham VAMC.

Specimens are fully de-identified (stripped of HIPAA 18 and study ID/code before being shared outside of Durham VAMC.

Data or specimens are coded and the code is not related to, or derived from, information about the individual and that code is not otherwise capable of being translated as to the identify the individual. Only someone possessing the key to code can link the data to a particular participant.

Other, specify:

1. **Suspected Loss of VA Information:**

Should any incident such as theft or loss of data, unauthorized access of sensitive data or non-compliance with security controls occur it will be immediately reported according to VA policy. All incidents regarding information security/privacy incidents will be reported to the ISO and PO within 1 hour of acknowledgement of issue and done so using the VHADUR Research Events Report e-mail group ([VHADURResearchEventReport@va.gov](mailto:VHADURResearchEventReport@va.gov)).

1. **Reporting of Results:**

Reporting of results, such as in scientific papers and presentations, will never identify individual subjects. Data will be presented in aggregate and individual-level data will not be published.

Other results reporting plan, describe:

1. **Future Use of Data:**

Data will be retained for future use. This is described elsewhere in the protocol and is noted in the HIPAA authorization.

Future Use of data is optional (i.e., not required by the research subject).

Future Use of data is required for participation in the study.

No future use of data is currently planned.

1. **Use of Mail Merge Technology**

Mail merge programs will be used to generate letters and/or address labels for mailings to potential or already enrolled research subjects. The study team is aware that to reduce risk of mail merge related privacy incidents, use of mail merge programs requires a 25% accuracy check to verify that (potential) research subject name and mailing address are properly “matched”. If discrepancies are found, a 100% accuracy check is required before letters may be mailed.

1. **Use of Non-Standard Software**

I do NOT intend to use any new specialized software (i.e. Software that’s not already approved OR installed) in this study.

I intend to use specialized software that has not already been installed and it has been approved for use by the VA Technical Reference Model (TRM) Group.

(Note: All new software must be approved by TRM before it can be installed on VA systems.)

I intend to use previously installed software on my VA computer.

1. **Use of Cloud Computing Services**

Cloud computing services will NOT be used in this study.

Cloud computing services WILL be used in this study as described below and have been approved nationally by the VA Chief Information Officer (CIO). (Note: ONLY cloud computing services that have been approved nationally may be used.)

**References**

Addicott MA, Luber B, Nguyen D, Palmer H, Lisanby SH, Appelbaum LG (2019). Low- and high-frequency repetitive transcranial magnetic stimulation effects on resting-state functional connectivity between the postcentral gyrus and the insula. *Brain Connectivity*, 9(4), 322-328.

Addicott MA, Sweitzer MM, Froelinger B, Rose JE, McClernon FJ (2015). Increased functional connectivity in an insula-based network is associated with improved smoking cessation outcomes. *Neuropsychopharmacol*, 40, 2640-2656.

American College of Obstetricians and Gynecologists. (2017). *ACOG Committee Opinion Number 721*. Retrieved March 3, 2020, from https://www.acog.org/-/media/Committee-Opinions/Committee-on-Obstetric-Practice/co721.pdf?dmc=1&ts=20170922T0723475369

Amiaz R, Levy D, Vainiger D, Grunhaus L, Zangen A (2009). Repeated high-frequency transcranial magnetic stimulation over the dorsolateral prefrontal cortex reduces cigarette craving and consumption. *Addiction*, 104, 653-660.

Awiszus F & Borckardt J (2011). *TMS Motor Threshold Assessment Tool 2.0 (MTAT 2.0*). Retrieved Feb 22, 2020, from https://www.clinicalresearcher.org/software.htm

Beck AT, Steer RA, Brown GK (1996). Manual for the Beck Depression Inventory-II. San Antonio, TX: Psychological Corporation.

Beckham JC, Calhoun PS, Dennis MF, Wilson SM, Dedert EA (2013). Predictors of lapse in first week of smoking abstinence in PTSD and non-PTSD smokers. *Nicotine Tob Res,* 15, 1122-9.

Beckham JC, Adkisson KA, Hertzberg J, et al. (2018). Mobile contingency management as an adjunctive treatment for co-morbid cannabis use disorder and cigarette smoking. *Addict Behav*, 79, 86-92.

Benowitz NL, Hukkanen J, Jacob P (2009). Nicotine chemistry, metabolism, kinetics and biomarkers. *Handb Exp Pharmacol,* 29-60.

Beynel L, Appelbaum LG, Luber B, et al. (2019). Effects of online repetitive transcranial magnetic stimulation (rTMS) on cognitive processing: A meta-analysis and recommendations for future studies. *Neurosci Biobehav Rev*, 107, 47-58.

Beynel L, Powers JP, Appelbaum LG. (2020). Effects of repetitive transcranial magnetic stimulation on resting-state connectivity: A systematic review. *Neuroimage*, 211, 116596.

Bikson M, Hanlon CA, Woods AJ, et al. (2020). Guidelines for TMS/tES clinical services and research through the COVID-19 pandemic. *Brain Stimulation*, 13(4), 1124-1149.

Blevins CA, Weathers FW, Davis MT, et al. (2015). The posttraumatic stress disorder checklist for *DSM-5* (PCL-5): development and initial psychometric evaluation. *J Trauma Stress*, 28, 489-498.

Bohn MJ, Babor TF, Kranzler HR (1991). Validity of the drug abuse screening test (DAST-10) in inpatient substance abusers: problems of drug dependence. National Institute of Drug Abuse Research Monograph 119, p. 223. Rockville, MD: Department of Health and Human Services. World Health Organization (WHO).

Bovin MJ, Marx BP, Weathers FW, et al. (2015). Psychometric properties of the PTSD checklist for diagnostic and statistical manual of mental disorders-fifth edition (PCL-5) in Veterans. *Psychol Assess*, 28, 1379-1391.

Centers for Disease Control and Prevention. (2002). Annual smoking-attributable mortality years of potential life lost and economic costs--United States, 1995-1999. *Morbidity and Mortality Weekly Report,* 51, 300-303.

Centers for Disease Control and Prevention. (2008). Smoking-attributable mortality, years of potential life lost, and productivity losses - United States, 2000-2004. *Morbidity and Mortality Weekly Report,* 57, 1226-1228.

Centers for Disease Control and Prevention. (2010). *Tobacco use: Targeting the nation's leading killer*. Atlanta, GA: Centers for Disease Control and Prevention.

Chang D, Zhang J, Peng W, et al. (2018). Smoking cessation with 20 Hz repetitive transcranial magnetic stimulation (rTMS) applied to two brain regions: a pilot study. *Front Hum Neurosci*, 12, 344.

Cole EJ, Stimpson KH, Bentzley BS, et al. (2020). Stanford accelerated intelligent neuromodulation therapy for treatment-resistant depression. *Am J Psychiatry*, 177(8), 716-726.

Colton CW & Manderscheid RW (2006). Congruencies in increased mortality rates, years of potential life lost, and causes of death among public mental health clients in eight states. *Prev Chronic Dis*, 3(2), A42.

Cook BL, Wayne GF, Kafali EN, et al. (2014). Trends in smoking among adults with mental illness and association between mental health treatment and smoking cessation. *JAMA,* 311, 172-82.

Counter SA & Borg E (1992). Analysis of the coil generated impulse noise in extracranial magnetic stimulation. *Electroencephalogr Clin Neurophysiol*, 85(4), 280-8.

Daskalakis ZJ, Moller B, Christensen BK, et al. (2006). The effects of repetitive transcranial magnetic stimulation on cortical inhibition in healthy human subjects. *Exp Brain Res*, 174, 403-412.

Dieler AC, Dresler T, Joachim K, et al. (2014). Can intermittent theta burst stimulation as add-on to psychotherapy improve nicotine abstinence? Results from a pilot study. *Eur Addict Res*, 20, 248-253.

Dinur-Klein L, Dannon P, Hadar A, et al. (2014) Smoking cessation induced by deep repetitive transcranial magnetic stimulation of the prefrontal and insular cortices: a prospective, randomized controlled trial. *Biol Psychiatry*, 76, 742-749.

Devilly GJ & Borkovec TD (2000). Psychometric properties of the credibility/expectancy questionnaire. *Journal of Behavior Therapy and Experimental Psychiatry*, 31, 73–86.

Ekhtiari H, Tavakoli H, Addolorato G, et al. (2019). Transcranial Electrical and Magnetic Stimulation (tES and TMS) for Addiction Medicine: A consensus paper on the present state of the science and the road ahead. *Neuroscience and Biobehavioral Reviews*, 104, 118-140.

Eichhammer P, Johann M, Kharraz A, et al. (2003). High-frequency repetitive transcranial magnetic stimulation decreases cigarette smoking. *J Clin Psychiatry*, 64, 951-953.

Fiore MC, Bailey WC, Cohen SJ, et al. *Treating Tobacco Use and Dependence. Clinical Practice Guideline.* Rockville, MD: U.S. Department of Health and Human Services, 2000.

First MB, Williams JBW, Karg RS, Spitzer RL (2015). *Structured Clinical Interview for DSM-5 (SCID-5 for DSM-5)*. Arlington, VA: American Psychiatric Association.

Food and Drug Administration (2015, March 9). *FDA Drug Safety Communication: FDA updates label for stop smoking drug Chantix (varenicline) to include potential alcohol interaction, rare risk of seizures, and studies of side effects on mood, behavior, or thinking* [Press Release]. Retrieved from https://www.fda.gov/drugs/drug-safety-and-availability/fda-drug-safety-communication-fda-updates-label-stop-smoking-drug-chantix-varenicline-include#:~:text=%5B3%2D9%2D2015%5D,with%20Chantix%20have%20been%20reported.

Fox MD, Halko MA, Eldaief MC, Pascual-Leone A (2012). Measuring and manipulating brain connectivity with resting state functional connectivity magnetic resonance imaging (fcMRI) and transcranial magnetic stimulation (TMS). *NeuroImage*, 62(4), 2232-2243.

Fu S, McFall M, Saxon AJ, et al. (2007). Posttraumatic stress disorder and smoking: A systematic review. *Nicotine Tob Res,* 9, 1071-84.

Globe Newswire (2020, August 24). *BrainsWay receives FDA clearance for smoking addiction in adults* [Press Release]. Retrieved from https://www.globenewswire.com/news-release/2020/08/24/2082476/0/en/ BrainsWay-Receives-FDA-Clearance-for-Smoking-Addiction-in-Adults.html

Greenberg G & Hoff R (2016). *2016 veterans with PTSD data sheet: National, VISN, and VAMC tables.* West Haven, CT. Center, N.P.E.

Hallet M (2000). Transcranial magnetic stimulation and the human brain. *Nature*, 405, 147-150.

Hallet M (2007). Transcranial magnetic stimulation: a primer. *Neuron*, 55, 187-199.

Hapke U, Schumann A, Rumpf H-J, et al. (2005). Association of smoking and nicotine dependence with trauma and posttraumatic stress disorder in a general population sample. *J Nerv Ment Dis*, 193(12), 843-846.

Hughes JR (2012). Background on the Minnesota Nicotine Withdrawawl Scale-Revised (MNSW-R). Retrieved March 5, 2020, from https://www.med.uvm.edu/behaviorandhealth/research/minnesota-tobacco-withdrawal-scale

Hauer L, Scarano GI, Brigo F, et al. (2019). Effects of repetitive transcranial magnetic stimulation on nicotine consumption and craving: A systematic review. *Psychiatry Research*, 281, 112562.

Heatherton TF, Kozlowski LT, Frecker RC, Fagerstrom KO (1991). The Fagerstrom Test for Nicotine Dependence: a revision of the Fagerstrom Tolerance Questionnaire. *Br J Addict*, 86, 1119-27.

Janes AC, Gilman JM, Radoman M, et al. (2017). Revisiting the role of the insula and smoking cue-reactivity in relapse: a replication and extension of neuroimaging findings. *Drug Alcohol Depend*, 179, 8-12.

Jonk YC, Sherman SE, Fu SS, et al. (2005). National trends in the provision of smoking cessation aids within the Veterans Health Administration. *Am J Managed Care,* 11, 77-85.

Joseph AM, McFall M, Saxon AJ, Chow BK, Leskala J, Dieperink ME, Carmody TP, Beckham JC. Smoking intensity and severity of specific symptom clusters in posttraumatic stress disorder (2012). *Journal of Traumatic Stress*, 25, 10-16.

Keel JC, Smith MJ, Wassermann EM (2001). A safety screening questionnaire for transcranial magnetic stimulation. *Clin Neurophysiol*, 112(4), 720.

Khedr EM, Tony AA, Abdelwarith A, Safwat M (2020). Effect of chronic nicotine consumption on motor cortical excitability: a transcranial magnetic stimulation study. J *Clin Neurophysiol*, 50(1), 33-39.

Krebs P, Rogers E, Smelson D, et al. (2018). Relationship between tobacco cessation and mental health outcomes in a tobacco cessation trial. *J Health Psychol*, 23(8), 1119-1128.

Li X, Hartwell KJ, Henderson, et al. (2020). Two weeks of image-guided left dorsolateral prefrontal cortex repetitive transcranial magnetic stimulation improves smoking cessation: A double-blind, sham-controlled, randomized clinical trial. *Brain stimulation*, *13*(5), 1271–1279.

Li X, Hartwell KJ, Owens M, et al. (2013). Repetitive transcranial magnetic stimulation of the dorsolateral prefrontal cortex reduces nicotine cue craving. *Biol Psychiatry*, 73, 714-720.

Little MA, Ebbert JO (2016). The safety of treatments for tobacco use disorder. *Expert Opinion on Drug Safety*, 15(3), 333-347.

McFall M, Saxon A, Malte CA, et al. (2010). Integrating tobacco cessation into mental health care for posttraumatic stress disorder: A randomized controlled trial. *JAMA,* 304, 2485-93.

Middleton EJ & Morice H (2000). Breath carbon monoxide as indication of smoking habit. *Chest*, 117, 758-763.

Miller D, Kalman D, Ren X, et al. (2001). *Health behaviors of veterans in the VHA: Tobacco abuse: 1999 large health survey of VHA enrollees*: Office of Quality and Performance: Department of Veterans Affairs.

Mokdad A, Marks J, Stroup D, Gerberding J (2004). Actual causes of death in the United States. *Journal of the American Medical Association*, 291, 1238-1245.

Naqvi NH & Bechara A (2010). The insula and drug addiction: an interoceptive view of pleasure, urges, and decision-making. *Brain Struct Funct*, 214(0), 435-450.

Naqvi NH, Rudrauf D, Damasio H, Bechara A (2007). Damage to the insula disrupts addiction to cigarette smoking. *Science*, 315(5811), 531-4.

National Imaging Tools and Resources Collaboratory (NITRC). *Artifact Detection Tools (ART)* (n.d.). Retrieved March 3, 2020, from https://www.nitrc.org/projects/artifact_detect

National Library of Medicine (NLM). *ClinicalTrials.gov* (n.d.). Retrieved March 3, 2020, from https://clinicaltrials.gov/ct2/results?cond=Smoking&term=tms&cntry=US&state=&city=&dist=&Search=Search

Pripfl J, Tomova L, Riecansky L, Lamm C (2014). Transcranial magnetic stimulation of the left dorsolateral prefrontal cortex decreases cue-induced nicotine craving and EEG delta power. *Brain Stimul*, 7, 226-233.

Prochaska JJ (2010). Failure to treat tobacco use in mental health and addiction treatment settings: a form of harm reduction? *Drug Alcohol Depend*, 110(3), 117-82.

Prochaska JJ, Das S, Young-Wolff KC (2017). Smoking, mental illness, and public health. *Annu Rev Public Health*, 38, 165-185.

Prochaska JJ, Delucchi K, Hall SM (2004). A meta-analysis of smoking cessation interventions with individuals in substance abuse treatment or recovery. *J Consult Clin Psychol*, 72(6), 1144-56.

Rose JE, McClernon FJ, Froelinger B, Behm FM, Preud’Homme X, Krystal AD (2011). Repetitive transcranial magnetic stimulation of the superior frontal gyrus modulates cravings for cigarettes. *Biol Psychiatry*, 70, 794-799.

Rossi S, Hallett M, Rossini PM, Pascual-Leone A, and the Safety of TMS Consensus Group (2009). Safety, ethical considerations, and application guidelines for the use of transcranial magnetic stimulation in clinical practice and research. *Clin Neurophysiol*, 120, 2008–2039.

Rossi, S., Antal, A., Bestmann, S. et al. (2021) Safety and recommendations for TMS use in healthy subjects and patient populations, with updates on training, ethical and regulatory issues: Expert guidelines. *Clin Neurophysiol,* 132, 269-306*.*

Rush, A.J., Carmody, T., & Reimitz, P. (2006). The Inventory of Depressive Symtomatology (IDS): Clinician (IDS-C and Self-Report (IDS-SR) ratings of depression symptoms. *Intl J Methods Psychiatr Res*, 9, 45-59.

Sammet S (2016). Magnetic resonance safety. *Abdominal Radiology*, 41(3), 444-451.

Smith SM, Goldstein RB, Grant BF (2016). The association between posttraumatic stress disorder and lifetime DSM-5 psychiatric disorders among veterans: Data from the National Epidemiologic Survey on Alcohol and Related Conditions-III (NESARC-III). *J Psychiatr Res*, 82, 16-22.

Sokal J, Messias E, Dickerson FB, et al. (2004). Comorbidity of medical illness who are receiving community psychiatric services. *J Nerv Ment Dis*, 192(6), 421-7.

Taylor G, McNeill A, Girling A, et al. (2014). Change in mental health after smoking cessation: Systematic review and meta-analysis. *BMJ*, 348, g1151.

Trojak B, Meille V, Achab S, et al. (2015). Transcranial magnetic stimulation combined with nicotine replacement therapy for smoking cessation: a randomized controlled trial. *Brain Stimul*, 8, 1168-1174.

Velicer WF, Friedman RH, Fava JL, et al. (2006). Evaluating nicotine replacement therapy and stage-based therapies in a population-based effectiveness trial. *Journal of Consulting and Clinical Psychology*, 74, 1162-1172.

Veterans Health Administration, DoVA & Epidemiology Program, P-DHG, Office of Patient Care Services. *Analysis of VA Health Care Utilization among Operation Enduring Freedom, Operation Iraqi Freedom, and Operation New Dawn Veterans*, *from 1st Qtr FY 2002 through 3rd Qtr FY 2015,* 2017.

Wagner T, Valero-Cabre A, Pascual-Leone A (2007). Noninvasive human brain stimulation. *Annu Rev Biomed Eng*, 9, 527-565.

Wassermann EM, Grafman J, Berry C, et al. (1996). Use and safety of a new repetitive transcranial magnetic stimulator. *Electroencephalogr Clin Neurophysiol*, 101, 412–7.

Weathers FW, Blake DD, Schnurr PP, et al. (2013). The Clinician-Administered PTSD Scale for DSM-5 (CAPS-5). [Assessment] Available from www.ptsd.va.gov

Wellcome Centre for Human Neuroimaging, University College London (2020). *Statistical parametric mapping software, SPM12.* Retrieved March 3, 2020, from https://www.fil.ion.ucl.ac.uk/spm/software/spm12/

Whitfield-Gabrieli S & Nieto-Castanon A (2012). Conn: a functional connectivity toolbox for correlated and anticorrelated brain networks. *Brain Connect*, 2(3), 125-41.

Woodward M & Tunstall-Pedoe H (1992). An interactive technique for identifying smoking deceivers with application to the Scottish heart health study. *Preventive Medicine*, 21, 88-97.
